# Supplementary material for: Phase 1, randomized, crossover study comparing intravenous GTX-104 to oral nimodipine in healthy human subjects
Source: PLoS One. 2025 Dec 8;20(12):e0323162. doi: 10.1371/journal.pone.0323162 (PMC12685190; doi:10.1371/journal.pone.0323162)
Supplement: S1 File — (DOCX) [file pone.0323162.s001.pdf]

16.1 STUDY INFORMATION

16.1.1 Protocol and Protocol Amendments

Note to file – dated 2021/11/30

Protocol Amendment 06

Memo

Acasti Pharma Inc.	
To	Study File Protocol GTX-104-002<br>(Altasciences Project GRT-P9-594)
From	Florence Clark, Director Clinical Operations
CC:	Acasti Pharma Master File
Date:	11-30-2021
Re:	Interim Analysis
Comments:	This note to file is to document that the interim analysis planned in the protocol after the first 20 subjects (Cohort 1 and 2) was completed. Based on the results, the decision was made to proceed at the same dose and infusion rate for the intravenous administration of GTX-104.<br><br>The following documents support the interim analysis:<br>***anova_ms*** (biostatistical results)<br>***GTX_BE-11192021_Delivered*** (power point presentation)<br>***GTX104 Acasti PK data-20211118*** (Individual PK parameters)<br>***GRT_P9_594_20211116_pk_info*** (dataset for PK parameters calculations)<br><br>Accordingly, the clinical trial will proceed as scheduled for Cohorts 3,4, 5 and 6. Altasciences (CRO) was notified on this decision on November 22, 2021.

Signature:

Florence Clark
Director, Clinical Operations

Signature:

Jean-François Lapointe
Sr. Director, Clinical Affairs

Signature:

Pierre Lemieux, Ph.D.
COO and CSO

CLINICAL TRIAL PROTOCOL

A Phase 1, Randomized, Two-Period Crossover Study to Evaluate the Relative Bioavailability of Intravenous GTX-104 Compared to Oral Nimodipine Capsules at Steady State in Healthy Male and Female Subjects

IND Number:	129969
Protocol Number:	GTX-104-002
Phase:	Phase 1
Investigational Drug:	GTX-104 (nimodipine for intravenous infusion)
Sponsor:	Grace Therapeutics, Inc.<br>685 US Highway One<br>Biotechnology Development Center, 2nd Floor<br>North Brunswick, NJ 08902
Principal Investigator:	Éric Sicard, M.D<br>Altasciences, 1200 Beaumont Ave., Mount-Royal, Quebec
Medical Monitor	Judith Johnson<br>Safe Harbor Pharmacovigilance, LLC<br>5205 Indigo Moon Way, Raleigh NC 27613.<br>Tel. (978) 618-7770.
Date:	03 May 2019 (Original Protocol)<br>07 August 2019 (Amendment 1)<br>29 September 2020 (Amendment 2)<br>11 August 2021 (Amendment 3)<br>13 August 2021 (Amendment 4)<br>20 August 2021 (Amendment 5)<br>22 September 2021 (Amendment 6)

GCP Statement

This study will be performed in compliance with Good Clinical Practice (GCP), the Declaration of Helsinki in its most recent form, and all federal, state, and local legal and regulatory requirements

CONFIDENTIAL STATEMENT

The information contained in this document and all information provided related to GTX-104 ("Study Drug") are the confidential and proprietary information of Grace Therapeutics (Sponsor) and except as may be required by federal, state, or local laws or regulations, may not be disclosed to others without prior written permission of the Sponsor. The Principal Investigator may, however, disclose such information to supervised individuals working on the Study Drug, provided such individuals agree to maintain the confidentiality of such information.

DocuSign Envelope ID: 5C33DBCA-0804-434B-BEF6-5BBB6BC0F6BF

PROTOCOL APPROVAL PAGE: SPONSOR SIGNATURE

Study Title: A Phase 1, Randomized, Two-Period Crossover Study to Evaluate the Relative Bioavailability of Intravenous GTX-104 Compared to Oral Nimodipine Capsules at Steady State in Healthy Male and Female Subjects

Protocol Number: GTX-104-002

Date of Issue: 03 May 2019 (Original Protocol)

07 August 2019 (Amendment 1)

29 September 2020 (Amendment 2)

11 August 2021 (Amendment 3)

13 August 2021 (Amendment 4)

20 August 2021 (Amendment 5)

22 September 2021 (Amendment 6)

Sponsor Name and Address:
Grace Therapeutics, Inc.
685 US Highway One
Biotechnology Development Center, 2nd Floor
North Brunswick, NJ 08902

This clinical trial protocol and amendments were subject to critical review and have been approved by the Sponsor. It is confirmed that the information and guidance given in this protocol and protocol amendments complies with scientific principles, the guidelines of Good Clinical Practices, the Declaration of Helsinki in the latest relevant version, and the applicable legal and regulatory requirements.

Sponsor Signatory:

________________________________ ________________

S. George Kottayil, Ph.D. Date
CEO
Grace Therapeutics, Inc.

PROTOCOL AGREEMENT: INVESTIGATOR SIGNATURE

Protocol Title: A Phase 1, Randomized, Two-Period Crossover Study to Evaluate the Relative Bioavailability of Intravenous GTX-104 Compared to Oral Nimodipine Capsules at Steady State in Healthy Male and Female Subjects

Protocol Number: GTX-104-002

Original Protocol (03 May 2019)

Amendment 1 (07 August 2019)

29 September 2020 (Amendment 2)

11 August 2021 (Amendment 3)

13 August 2021 (Amendment 4)

20 August 2021 (Amendment 5)

22 September 2021 (Amendment 6)

I agree to conduct the trial as outlined in the protocol amendments and in accordance with the Sponsor's guidelines and Good Clinical Practice requirements. I agree to maintain the confidentiality of all information received or developed in connection with this protocol.

______________________________ ____________

Investigator's Signature Date

Éric Sicard, M.D
____
Print Name

SYNOPSIS

**Protocol Title**	A Phase 1, Randomized, Two-Period Crossover Study to Evaluate the Relative Bioavailability of Intravenous GTX-104 Compared to Oral Nimodipine Capsules at Steady State in Healthy Male and Female Subjects
**Protocol Number**	GTX-104-002
**Phase**	1
**Study Design & Participant Duration**	This is a Phase 1, single center, randomized, two-period crossover study in healthy male and female subjects designed to evaluate the relative bioavailability (BA) and safety at steady state of two formulations of nimodipine: GTX-104 (nimodipine for intravenous [IV] infusion; test formulation) and nimodipine oral capsules, RS (reference formulation).<br>After a 45-day Screening period where eligibility will be assessed, healthy male and female subjects will be admitted to the clinical research unit (CRU) on the day prior to dosing (Day -1) and remain domiciled in the CRU for the duration of each study period. At admission, subjects will be randomly assigned in a 1:1 ratio to 1 of 2 treatment sequences: AB or BA, where Treatment A and Treatment B are as follows:<br>• Treatment A (GTX-104, Test): Nimodipine will be administered by infusion over 72 hours. Administration will be a 30-minute infusion of 4 mg given every 4 hours (q4h) in addition to a continuous infusion of 0.15 mg/h given for 72 hours. The total drug infused over the first 30 minutes of each 4-hour dosing interval will equal 4.075 mg and the total drug infused every 4 hour dosing interval will be 4.6 mg.<br>• Treatment B (NIMOTOP® RLD): Nimodipine capsules (RS) administered orally with 240 mL of water at a dose level of 60 mg (two 30 mg capsules) q4h for 72 hours.<br>Starting on Day 1, subjects will receive each treatment in the order dictated by their assigned sequence with each treatment period separated by a minimum of 96 hours to allow for treatment washout.<br>On Days 1 to 4 of each treatment period, study drug will be administered at the same time each day (starting at approximately 8 AM on Day 1). Each morning (4 AM and 8 AM) doses will be administered in fasted status, and breakfast will be provided 2 hours after the 8 AM dose (e.g., subjects should be fasted from between 2 AM to 10 AM). Standardized meals and snacks will then be provided at approximately uniform times throughout the day between 10 AM to 2 AM of the next day, such that the 4 AM and 8 AM doses (e.g., the oral dose or the q4h infusion for GTX-104) are administered to subjects in the fasted state and other doses of 12 PM, 4 PM, 8 PM and 12 AM will be administered in fed status. On Days 3 and 10 only, water will be restricted 30 minutes before and 1 hour after administration of both IV and oral doses. Outside of water administered with study drug, and 30 minutes before and 1 hour after Days 3 and 10 doses (IV and oral) water will be allowed *ad libitum.*<br>Blood samples for analysis of nimodipine pharmacokinetics (PK) will be taken at the following timepoints:<br><u>Treatment A (Nimodipine Infusion):</u><br>*Note: PK and clinical venous blood sampling must occur in the arm opposite to infusion, and the infusion line should not be used for PK sampling.*<br>• Day 1: Pre-dose (time 0, before initiation of the first [8 AM]

	infusion), and at 0.33, 0.5, 0.75, 1, 1.33, 2, 3, and 4 hours post-dose.<br>• Starting on Day 3, blood samples for PK should be taken at: 48 (prior to initiation of the 8 AM 4 mg dose), 48.33, 48.5, 48.75, 49, 49.33, 50, 51, 52, 52.33, 52.5, 52.75, 53, 53.33, 54, 55, 56, 56.33, 56.5, 56.75, 57, 57.33, 58, 59, 60, 60.33, 60.5, 60.75, 61, 61.33, 62, 63, 64, 64.33, 64.5, 64.75, 65, 65.33, 66, 67, 68, 68.33, 68.5, 68.75, 69, 69.33, 70, 71, 72-hours post-start of the first infusion.<br>• Note, for timepoints that are scheduled at the same time as the 4 mg dose (48, 52, 56, 60, 64, 68, and 72 hours post-start of infusion), blood for PK analysis should be taken before initiation of the 30-minute infusion. For the 48.5, 52.5, 56.5, 60.5, 64.5, and 68.5-hour timepoints, blood samples for PK analysis should be collected after completion of the 4 mg infusion.<br>Treatment B (Oral Nimodipine):<br>*Note: Subjects will take a total of 18 oral doses during the 72 hours of treatment, administered q4h starting at approximately 8 AM on Day 1 (e.g., 8 AM, 12 PM, 4 PM, 8 PM, 12 AM, 4 AM).*<br>• Day 1: Pre-dose (time 0, before the 1st [8 AM] dose), and at 0.33, 0.5, 0.75, 1, 1.33, 2, 3, and 4 hours post-dose.<br>• Day 3, blood samples for PK should be taken at: 48 (prior to initiation of the 8 AM 60 mg dose), 48.33, 48.5, 48.75, 49, 49.33, 50, 51, 52, 52.33, 52.5, 52.75, 53, 53.33, 54, 55, 56, 56.33, 56.5, 56.75, 57, 57.33, 58, 59, 60, 60.33, 60.5, 60.75, 61, 61.33, 62, 63, 64, 64.33, 64.5, 64.75, 65, 65.33, 66, 67, 68, 68.33, 68.5, 68.75, 69, 69.33, 70, 71, 72-hours since the first dose.<br>• Note, for timepoints that are scheduled at the same time as the 60 mg oral dose (48, 52, 56, 60, 64, 68, and 72 hours since first dose), blood for PK analysis should be taken before oral dose intake.<br>Safety assessments will be collected throughout the study and will include concomitant medications, adverse events (AEs) and serious AEs (SAEs), 12-lead electrocardiograms (ECGs), clinical laboratory evaluations, and resting vital signs (including blood pressure [BP]). During Treatment A and Treatment B, subjects will have BP recorded at baseline (prior to first dose) with an oscillometric sphygmomanometer and 60 minutes after dosing for the 8:00 AM, 12:00 PM, 4:00 PM, and 8:00 PM doses on Days 3 and 10 only.<br>After the final PK sample of treatment period 2, subjects will undergo End of Study (EOS) assessments and be discharged from the CRU, if safety parameters are acceptable to the Investigator.<br>The study will be defined by a Screening Period (45 days), CRU admission, and two 72-hour treatment periods separated by a minimum 96-hour washout period (total 12 days in the CRU assuming the minimum washout period).<br>Participant duration in this study, from Screening to EOS assessments, is expected to be approximately 56 days.
**Study Objectives**	The primary objective of this study is to:<br>• Evaluate the relative bioavailability of GTX-104 administered by IV infusion versus nimodipine oral capsules at steady state.<br>The secondary objective of the study is to:<br>• Assess the safety and tolerability of GTX-104 and nimodipine oral capsules in healthy subjects.

**Study Endpoints**	Primary:<br>The following nimodipine PK parameters will be the primary endpoints:<br>• Maximum concentration (C_{max}) on Day 1 for the first dose (8 AM dose)<br>• Area under the concentration-time curve (AUC) on Day 3 ($AUC_{Day\ 3,\ 0\text{-}24hr}$) from 8:00 AM to 8:00 AM<br>Secondary:<br>Additional nimodipine PK parameters include:<br>• C_{max} on Day 3 across all 6 doses<br>• Absolute bioavailability (F) of oral nimodipine capsules (Nimotop®), RLD<br>• Total body clearance of the drug from plasma after IV infusion (CL)<br>• Apparent total clearance of the drug from plasma after oral administration (CL/F)<br>Safety:<br>Safety endpoints will include:<br>• Incidence of treatment-emergent AEs (TEAEs) and SAEs, grouped by system organ class, preferred term, relationship to study drug, severity, and treatment regimen<br>• Change from baseline in clinical laboratory evaluations (hematology, clinical chemistry, and urinalysis), vital signs, physical examination results, and 12-lead safety ECG parameters at the end of the study
**Number of Subjects**	Planned: Minimum of 60 subjects enrolled, to ensure at least 50 completing subjects
**Number of Sites & Participating Countries**	This trial will take place at one site in Canada.
**Subject Population**	The overall study population will be healthy male and female adult subjects, ages 18 to 55 years, inclusive.
**Key Inclusion & Exclusion Criteria**	**Subject Inclusion Criteria**<br>Subjects are eligible for inclusion only if all of the following criteria are met:<br>1. An IRB-approved informed consent form is signed and dated prior to any study-related activities.<br>2. Subject is 18 to 55 years of age, inclusive, at time of consent.<br>3. Subject has a body mass index (BMI) between 18 and 32 kg/m^2, inclusive.<br>4. Negative COVID-19 virus test within 5 days of CRU admission.<br>5. Subject is able to communicate clearly with the Investigator and study staff, and willing and able to understand and follow instructions, comply with the

	protocol requirements, and make all required study visits.<br>6. Subject is in good general physical health as determined by absence of clinically significant medical or psychiatric history, physical examination findings, vital signs, clinical laboratory evaluations, and 12-lead ECG measurements.<br>• The 12-lead ECG should be consistent with normal cardiac conduction and function at Screening, including ventricular rate between ≥45 and ≤100 beats per minute (bpm), a corrected QT (QTc) interval ≤450 ms for male subjects or ≤470 ms for female subjects; QRS interval <120 ms; PR interval <220 ms; and morphology consistent with healthy conduction and function. 12-lead ECGs may be repeated once, at the discretion of the Investigator.<br>7. Subject has vital signs at Screening that are stable (measured in sitting or semi-reclined position after at least 5 minutes of rest) and are within the following ranges:<br>• Systolic blood pressure (SBP): ≥100 and ≤150 mmHg<br>• Diastolic blood pressure (DBP): ≥55 and ≤100 mmHg<br>• Heart rate: ≥45 and ≤100 bpm<br>Note: If vital signs are out-of-range, the Investigator may obtain one additional reading so that up to 2 consecutive assessments are made within 1 hour with the subject seated quietly during the 5 minutes preceding the assessment.<br>8. Subject is a nonsmoker (for at least 6 months) and does not use tobacco-containing products (including but not limited to, cigarettes, pipes, cigars, chewing tobacco, or nicotine patch or gum).<br>9. Subject has not consumed and agrees to abstain from taking any prescription drugs, (except as authorized by the Investigator **AND** Medical Monitor) for 7 days prior to first dose of study drug and continuing through EOS.<br>10. Subject has not consumed and agrees to abstain from taking dietary supplements including vitamins and herbal preparations, or non-prescription drugs

	(except as authorized by the Investigator **<u>AND</u>** Medical Monitor) for 30 days prior to first dose of study drug and continuing through EOS.<br>11. Subject has clinical chemistry, serology (screening only), hematology, coagulation (fasted), and complete urinalysis results at Screening and admission to the CRU within the reference range for the testing laboratory, unless the out-of-range results are deemed not clinically significant by the Investigator.<br>12. Subject has a negative urine drug screen at Screening and at CRU admission.<br>13. Subject is willing to submit a buccal swab or blood test for genetic testing to identify metabolic genotype for CYP3A4.<br>14. Female subjects only must have a negative pregnancy test (serum β-human chorionic gonadotropin [hCG]) at Screening and at CRU admission and must not be lactating.<br>15. Male subjects who are nonsterilized and sexually active with a female partner of childbearing potential and female subjects of childbearing potential must agree to use a protocol-approved method of contraception from signing of informed consent, throughout the duration of the study and for 30 days after the last dose of study drug.<br>16. Female subjects of nonchildbearing potential must be surgically sterile (i.e., hysterectomy, bilateral oophorectomy, or bilateral tubal ligation) and at least 6 months post-surgery, or must be menopausal, defined as no menstrual period for at least 12 months and confirmed by follicle-stimulating hormone (FSH) level of ≥40 IU/L.<br>17. Male subjects only must agree to not donate sperm during the study and for at least 30 days after their final dose of study drug.<br>18. Subject has good bilateral venous access, as judged by the Investigator or designee.<br>Subject Exclusion criteria<br>Subjects are ineligible for the study if <u>any</u> of the following criteria are met:<br>1. Does not meet all inclusion criteria.<br>2. Positive test for COVID-19 virus. Subjects will be retested for COVID-19 virus with sample collected

	on Day 4. If Day 4 test is positive, subject will be discharge from the unit and withdrawn from the study.<br>3. History or presence of clinically significant medical illness, including, but not limited to, cardiovascular, pulmonary, hematologic, endocrine, immunologic, dermatologic, neurologic, psychiatric, renal, hepatic, chronic respiratory, or gastrointestinal disease, that could interfere with the interpretation of the study.<br>4. Has current or recent (within 6 months) history of gastrointestinal disease or any surgical or medical condition (e.g., Crohn's or liver disease) that could potentially alter the absorption, metabolism, or excretion of the study drug.<br>5. Has any medical condition, physical exam finding, out-of-normal-range laboratory value, or 12-lead ECG at Screening or admission that has not been reviewed, approved, and documented as not clinically significant by the Investigator.<br>6. Prior history of clinically significant abnormal ECG (e.g., second- or third-degree heart block, uncontrolled arrhythmia, QTcF [Fridericia's correction] interval >450 ms for male subjects or >470 ms for female subjects) or abnormal cardiovascular exam at Screening that does not fall within values given for SBP, DBP, and heart rate in the inclusion criteria.<br>7. Subject has symptomatic bradycardia, symptomatic hypotension, or any prior history of myocardial infarction.<br>8. Positive test for orthostatic hypotension.<br>9. History or presence of malignancy within the past 5 years, except for adequately treated localized skin cancer (basal cell or squamous cell carcinoma).<br>10. Suffers from clinically significant systemic allergic disease or has a history of significant drug allergies, including, but not limited to, a history of anaphylactic reactions, allergic reactions due to any drug leading to significant morbidity, or known hypersensitivity to any compound in the study products or related compounds.<br>11. Subject has donated >500 mL or more of blood (or had equivalent blood loss) within 3 months prior to drug administration or intends to donate blood (to

	blood bank) within 3 months after completion of the study.<br>12. Has had an acute, clinically significant illness within 30 days prior to first study drug administration on Day 1 or has had a recent febrile illness with an abnormal body temperature for at least 72 hours before dosing on Day 1.<br>13. Has a history (within 12 months before Screening) of illicit drug abuse or has positive test for drugs of abuse at any time.<br>14. Has a history (within 180 days of Screening) of alcohol abuse, defined as alcohol consumption of more than 2 drinks per day [maximum 14 drinks per week] for males or more than 1 drink per day [maximum 7 drinks per week] for females, and/or has a positive alcohol test at any time during the study, or is unwilling to abstain from alcohol throughout the study. One drink = 5 ounces of wine, 12 ounces of beer, or 1.5 ounces of hard liquor).<br>15. Has a smoking history during the past 6 months (calculated from first dosing), including the use of any nicotine-containing substances (e.g., nicotine patch or gum, chewing tobacco, e-cigarettes), or has a positive cotinine test at Screening or upon admission to the CRU, or is unwilling to abstain from these products for the duration of the study.<br>16. Has had treatment with an investigational drug or experimental medical device within 28 days before study drug administration or five half-lives of the study drug's elimination half-life, whichever is longer.<br>17. Subject is a known CYP3A4 intermediate or poor metabolizer, or screening genotyping indicates subject is a CYP3A4 intermediate or poor metabolizer.<br>18. Has used any of the following types of medications:<br>• Received a strong or moderate inhibitor of CYP3A4 within 14 days or 5 half-lives, whichever is longer, of dosing on Day 1.<br>• Received more than 1 dose of a CYP3A4 inducer within 30 days before admission to the CRU.<br>• Within 2 weeks before Day 1: Any therapy that is known to interfere with glucuronidation

	of drugs, or that is known to exacerbate renal dysfunction (e.g., amphotericin B, aminoglycoside antibiotics, trimethoprim, nonsteroidal anti- inflammatory drugs [NSAIDs]), or antihypertensive agents that excessively reduce cardiac output or systemic BP. Over-the-counter medications such as acetaminophen, topical medication, and nutritional and vitamin supplements will be allowed at the discretion of the Investigator.<br>• Within 30 days before Day 1: Oral contraceptives, hormone replacement therapy, or any estrogen-containing medications, any monoamine oxidase inhibitors (MAOIs), prescription medicines or herbal preparations (e.g., St John's wort), or received any immunizations.<br>• Within 3 months before Screening: Opioids, neuroleptics, lithium, antidepressants, mood stabilizers, benzodiazepines, cognitive enhancers, centrally acting antihypertensives, or γ-aminobutyric acid (GABA) agonists.<br>• Within 6 months before Screening: Any implanted or injected testosterone product; subject must also agree to not use testosterone replacement products (including, but not limited to, topical, nasal, sublingual, or oral testosterone products) for the duration of the study through completion of the EOS assessments.<br>• Has consumed grapefruit, pomelo, or Seville orange-containing foods or beverages within 7 days before admission to the CRU, or has consumed any products containing caffeine and/or xanthine within 72 hours prior to admission, or is unwilling to abstain from these products during the inpatient portion of the study.<br>19. If female, the subject is pregnant or lactating, or intends to become pregnant before, during, or within 30 days after participating in this study; or intends to donate ova during such time period.<br>20. If male, the subject intends to impregnate others, or donate sperm during the course of this study or within 30 days after participating in this study.

	21. Has inadequate venous access for the required blood draws for the study.<br>22. Is unable to meet or perform study requirements or has a known or suspected inability to comply with the study protocol.<br>23. Is unable or unwilling to eat provided food (e.g., vegetarian, kosher, lactose-intolerant).<br>24. Is an immediate family member of the Investigator, or an employee of the study center, with direct involvement in the proposed study, or other studies under the direction of the Investigator or study center, or is in a dependent relationship with a study center employee who is involved in the conduct of this study (e.g., spouse, parent, child, sibling), or may consent under duress.
**Investigational Product, Dose, and Mode of Administration**	GTX-104, nimodipine for IV infusion; GTX-104 will be administered as a 30-minute infusion of 4 mg q4h in addition to a continuous infusion of 0.15 mg/h for 72 hours
**Reference Therapy, Dose, and Mode of Administration**	Nimotop® (RLD), nimodipine capsule (RS) 60 mg (two 30 mg capsules) administered q4h; oral
**Sample Size Calculation**	A model-based approach was used to estimate power and sample size. Simulated subjects (n=1000) were randomized to a two-period crossover with 2 sequences (AB and BA) where Treatment A was GTX-104 and Treatment B was oral nimodipine. Incorporated sample collection times are outlined in the study design. Day 1 and Day 3 (steady state) simulated data were used for C_{max} on Day 1 and $AUC_{Day\ 3,\ 0\text{-}24hr}$ power analysis. The estimate for the within subject variance (on the log scale) came from a mixed effects model that included fixed effects for sequence, period, and treatment, and a random effect for subject. Under the hypothesis that the true geometric mean ratio (GMR) is 1 for GTX-104 compared to oral nimodipine, a total of 49 subjects was estimated to provide power of 0.85 (C_{max} on Day 1) and >0.99 ($AUC_{Day\ 3,\ 0\text{-}24hr}$) to assess relative bioavailability in a two-period, two-sequence crossover study.<br>A minimum of 60 subjects was therefore selected for enrollment to allow at least 50 subjects completing (allowing for some subject attrition).
**Analysis Populations**	Safety population: The Safety Population will consist of all subjects who receive at least 1 dose of study drug.<br>PK Population: The PK Population will consist of all subjects who receive at least 1 dose of study drug and have evaluable PK data.<br>Analysis Population: The Analysis Population will consist of those subjects in the PK population who are evaluable for both Treatment A (test) and Treatment B (reference).
**Pharmacokinetic Analysis**	Individual nimodipine PK parameters will be calculated using noncompartmental analyses and will include Day 1 C_{max} (C_{max} of first 8 AM dose on Day 1), Day 3 $AUC_{Day\ 3,\ 0\text{-}24hr}$, Day 3 C_{max} (max concentration of

	Day 3 for each of the 6 doses), CL (for IV), CL/F (for oral doses), and F as a minimum. Individual PK parameters will be summarized by treatment using descriptive statistics. The PK population will be used.
**Safety Analysis**	All safety data will be listed by subject. TEAEs will be summarized for each treatment by system organ class, preferred term, severity, and relationship to test article. Observed values and changes from baseline for clinical laboratory test data, safety ECGs, physical examination results, and vital signs will be summarized using appropriate descriptive statistics. The Safety Population will be used.
**Statistical Considerations**	To assess the relative BA of GTX-104 (Test) and nimodipine oral capsules, RS (Reference), an analysis of variance (ANOVA) model, with treatment, sequence, and period as fixed effects and subject nested within sequence as a random effect, will be performed on natural log-transformed $AUC_{Day\ 3,\ 0-24hr}$ and C_{max} (of first dose on Day 1) from both treatment periods. The differences in the least squares means and associated 90% confidence intervals (CIs) will be back-transformed to provide the geometric ratios (Test/Reference) along with the 90% CIs for the ratios. The Analysis Population will be used.<br>In addition to the above comparisons, the C_{max} of Day 3 for both treatments will be presented graphically; the geometric mean of C_{max} values of both treatments will also be compared for all 6 doses.

TABLE OF CONTENTS

CLINICAL TRIAL PROTOCOL 1
PROTOCOL APPROVAL PAGE: SPONSOR SIGNATURE 3
PROTOCOL AGREEMENT: INVESTIGATOR SIGNATURE 4
SYNOPSIS 5
Subject Inclusion Criteria 7
TABLE OF CONTENTS 15
TABLE OF TABLES....18
TABLE OF FIGURES....18
ABBREVIATIONS....19
1. INTRODUCTION 21
1.1. Background 21
1.2. GTX-104 22
1.2.1. Clinical Experience with GTX-104 22
1.2.2. Clinical Experience with Nimodipine....22
1.2.3. Potential Benefits and Assessment of Potential Risks 23
1.3. Rationale and Dose Justification....23
1.3.1. Safety Experience with Selected Dose Regimens....26
1.3.2. Dose Justification....27
2. STUDY OBJECTIVES 28
2.1. Primary Objective 28
2.2. Secondary Objective 28
3. STUDY ENDPOINTS 28
3.1. Primary Endpoints....28
3.2. Secondary Endpoints....28
3.3. Safety Endpoints 28
4. INVESTIGATIONAL PLAN 28
4.1. Overall Study Design 28
4.1.1. Study Schematic....31
4.1.2. Time and Events Schedule 34
4.2. Scientific Rationale for Study Design....36
4.3. Study Procedures....36
4.3.1. Screening (Study Day -45 to Day -2)....36
4.3.2. CRU Admission (Day -1)....36
4.3.3. Treatment Period (Day 1 to 11) 36
4.3.4. End of Study/Early Termination (Day 11)....37
4.4. Discontinuation Criteria for Subjects and Trial 37
4.5. Duration of Treatment....37
4.6. End of Study Definition 38

5. STUDY POPULATION 38
5.1. Inclusion Criteria 38
5.2. Exclusion Criteria 39
5.3. Screen Failures 42
5.4. Removal of Subjects 42
5.5. Replacement of Subjects 42
6. TREATMENT OF SUBJECTS 42
6.1. Description of Treatments 42
6.1.1. Investigational Product 42
6.1.2. Reference Product 43
6.2. Treatments Administered 43
6.3. Timing of Dose 43
6.3.1. Food and Water Intake 43
6.4. Randomization 44
6.5. Blinding 44
6.6. Restrictions and Prohibitions 44
6.6.1. Birth Control 44
6.6.2. Prior and Concomitant Therapy 45
6.6.3. Subject Activity 46
6.6.4. Treatment Compliance 46
7. STUDY DRUG MATERIALS AND MANAGEMENT 46
7.1. Packaging and Labeling 46
7.1.1. Investigational Product 46
7.1.2. Reference Product 47
7.2. Storage and Accountability 47
7.2.1. Investigational Product Storage 47
7.2.2. Reference Product Storage 47
7.2.3. Accountability 47
7.3. Study Drug Dispensing 48
7.4. Bioavailability Sample Retention 48
8. STUDY EVALUATIONS 48
8.1. Informed Consent 48
8.2. Subject Identification Number 49
8.3. Demographics and Medical History 49
8.4. Physical Examinations 49
8.4.1. Height, Weight, and Body Mass Index 49
8.5. Vital Signs 50
8.6. Orthostatic Hypotension 50
8.7. Electrocardiograms 50
8.8. CYP3A4 Genotyping 51

8.9. Clinical Laboratory Evaluations 51
8.9.1. Reference Ranges 52
8.9.2. Sample Collection and Storage 53
8.9.3. Laboratory Results Review 53
8.9.4. Good Laboratory Practice Compliance 53
8.10. Blood Pressure Monitoring 53
8.11. Pharmacokinetic Assessments 54
8.11.1. Pharmacokinetic Blood Sample Collection 54
8.11.2. Pharmacokinetic Assessment Windows 55
8.11.3. Bioanalytical Analysis 55
8.12. Safety 55
8.13. Concomitant Medication 55
8.14. Assessment Windows 55
8.15. Other Study Procedures 56
8.15.1. Nausea or Vomiting 56
8.15.2. Potential Pregnancies 56
8.15.3. Unplanned infusion interruptions: 56
9. ASSESSMENT OF SAFETY 57
9.1. Definitions 57
9.1.1. Adverse Event 57
9.1.2. Life-threatening Adverse Event 58
9.1.3. Serious Adverse Event 58
9.2. Recording 58
9.3. Severity 59
9.4. Clinical Laboratory Adverse Events 59
9.5. Timing 59
9.6. Follow-up 59
9.7. Relationship or Causality 59
9.8. Reporting Adverse Events 60
9.9. Stopping Rules 61
10. DATA ANALYSIS AND STATISTICAL CONSIDERATIONS 62
10.1. General Considerations 62
10.2. Determination of Sample Size 63
10.3. Analysis Populations 63
10.4. Demographics and Baseline Characteristics 63
10.5. Pharmacokinetic Analyses 63
10.5.1. Interim Pharmacokinetic Analysis 63
10.5.2. Final Pharmacokinetic Analysis 64
10.6. Safety Analysis 64

11. QUALITY CONTROL AND QUALITY ASSURANCE 64
11.1. Study Monitoring 64
11.2. Audits and Inspections 65
11.3. Case Report Forms and Study Records 65
11.4. Protocol Deviations 65
12. ADMINISTRATIVE CONSIDERATIONS 66
12.1. Ethics 66
12.1.1. Ethics Review Board Approval 66
12.1.2. Ethical Conduct of the Study 66
12.1.3. Subject Information and Consent 66
12.1.4. Confidentiality 66
12.2. Data Handling and Recordkeeping 67
12.2.1. Direct Access to Source Data/Documents 67
12.2.2. Inspection of Records 67
12.2.3. Retention of Records 67
12.3. Financial Disclosure 68
12.4. Publication and Disclosure Policy 68
13. REFERENCES 69
APPENDICES 72

TABLE OF TABLES

Table 1-1. Simulated PK Parameters for Nimodipine Administered as IV and Oral Doses 24
Table 4-1. Time and Events Schedule 34
Table 6-1. Prohibited Prior and Concomitant Treatments 45
Table 8-1. Clinical Laboratory Evaluations 52
Table 8-2. Action to be Taken for Infusion Interruptions 57
Table 9-1. Contact Information for SAE Reporting 60

TABLE OF FIGURES

Figure 1-1. Simulated Concentration-Time Profiles for IV and Oral Regimens on Day 1, First Dose at 8 AM. 25
Figure 1-2. Simulated Concentration-Time Profiles for IV and Oral Regimens on Day 3. 25
Figure 1-3. Overall Simulated Cmax on Day 3 for IV and Oral Regimens 26
Figure 4-1. Study Schematic 31
Figure 4-2. Study Drug Administration Schematic (Treatment A; GTX-104) 32
Figure 4-3. Study Drug Administration Schematic (Treatment B; Oral Nimodipine) 33

ABBREVIATIONS

ADR	Adverse drug reactions
AE	Adverse events
ALT	Alanine aminotransferase
ANOVA	Analysis of variance
aSAH	Aneurysmal SAH
AST	Aspartate aminotransferase
ATC	Anatomical therapeutic chemical
AUC	Area under the concentration-time curve
BA	Bioavailability
BMI	Body mass index
BP	Blood pressure
bpm	Beats per minute
CI	Confidence interval
CFR	Code of Federal Regulations
CL	Total body clearance of the drug from plasma after intravenous infusion
CL/F	Apparent total clearance of drug from plasma after oral administration
C_{max}	Maximum concentration
CRU	Clinical research unit
CS	Clinically significant
CSR	Clinical study report
CYP	Cytochrome P450
DBP	Diastolic blood pressure
DCI	Delayed cerebral ischemia
ECG	Electrocardiogram
eCRF	Electronic case report form
ECG	Electrocardiogram
EDC	Electronic data capture
EOS	End of Study
ET	Early termination
F	Absolute bioavailability
FDA	Food and Drug Administration
FSH	Follicle-stimulating hormone
GABA	γ-aminobutyric acid
GCP	Good Clinical Practice
GLP	Good Laboratory Practice
GMR	Geometric mean ratio
HbA1c	Hemoglobin A1c
HBsAG	Hepatitis B surface antigen
hCG	human chorionic gonadotropin
HCV	Hepatitis C virus
HIPAA	Health Insurance Portability and Accountability Act
HIV	Human immunodeficiency virus
ICF	Informed consent form
ICH	International Conference on Harmonisation
IND	Investigational New Drug
IRB	Institutional review board
IUD	Intrauterine device

IV	Intravenous
LC-MS/MS	Liquid chromatography with tandem mass spectrometry
MAOI	Monoamine oxidase inhibitors
MAP	Mean arterial pressure
MedDRA	Medical Dictionary for Regulator Activities
NCS	Not clinically significant
NONMEM	Nonlinear mixed effects model
NSAID	Nonsteroidal anti- inflammatory drugs
OTC	Over-the-counter
PK	Pharmacokinetics
PKAP	Pharmacokinetic analysis plan
popPK	population pharmacokinetic
q4h	Every 4 hours
QA	Quality Assurance
QTc	Corrected QT
QTcF	Fridericia's correction
RLD	Reference Listed Drug
RS	Reference Standard
RSE	Relative standard error
SAE	Serious adverse event
SAH	Subarachnoid hemorrhage
SAP	Statistical analysis plan
SBP	Systolic blood pressure
SD	Standard deviation
SID	Subject identification
TEAE	Treatment-emergent adverse event
TID	Three times a day
T_{max}	Time to maximum plasma concentration
ULN	Upper limit of normal
U.S.	United States
USP	United States Pharmacopeia
WHO	World Health Organization
VPC	Visual predictive check

1. INTRODUCTION

1.1. Background

Subarachnoid hemorrhage (SAH), caused primarily by rupture of an aneurysm, accounts for about 5% of all strokes and has an incidence of 6 per 100,000 person years. Approximately 10% to 15% of aneurysmal SAH (aSAH) patients die before reaching the hospital, and those who survive the initial hours and are admitted or transferred to tertiary care centers are at risk of complications, most important of which include rebleeding and delayed cerebral ischemia (DCI) (Rinkel, 2016). Poor outcome from aSAH occurs in approximately 70% of patients: half die within 1 month after the hemorrhage, and of those who survive the initial month, half remain permanently dependent on help with activities of daily living. Despite improvements in the clinical management of aSAH over the last decade, DCI remains the single most important cause of morbidity and mortality in patients who survive the initial bleed.

Nimodipine, a potent calcium antagonist with a preferential relaxant effect on cerebral vessels is approved in the United States (U.S.) for the improvement of neurological outcome by reducing the incidence and severity of ischemic deficits in adult patients with aSAH (Nimodipine Capsules [Drug Label Information], August, 2012). Nimodipine remains the only therapeutic intervention proven to improve functional outcomes after SAH (Allen et al., 1983; Barker et al., 1996; Diringer et al., 2011; Harders et al., 1996; Pickard et al., 1989; Rinkel, 2016; Rowland et al., 2012).

Enteral nimodipine in the form of oral capsules (NIMOTOP®; NIMOTOP (nimodipine) capsules for oral use [Approved Label], 2005) and as an oral solution (NYMALIZE®; NYMALIZE (nimodipine) oral solution [Prescribing Information], 2013) is accepted as the standard of care in the U.S. in patients with aSAH (Dorhout Mees et al., 2007). Medication of obtunded patients by oral administration of crushed tablets or dilutions of the solution administered via nasogastric tube, jejunostomy, gastrostomy, or other feeding tube is a method that is unreliable, inexact, and can be complicated by aspiration or g-tube malfunctions. Also, the results of several pilot studies indicate that the rate and extent of nimodipine absorption from the gastrointestinal tract following enteral administration in some acute SAH patients could be negligible, and this may particularly be the case in patients with a decreased level of consciousness (Abboud et al., 2015; Soppi et al., 2007; Vinge et al., 1986).

An intravenous (IV) infusion solution formulation of nimodipine (NIMOTOP®; NIMOTOP (0.02% Solution for Infusion) [Summary of Product Characteristics], July 2016) is not approved in the U.S. but is approved in Europe for aSAH. It provides greater bioavailability, more stable plasma concentrations, and easier maintenance of controlled blood pressure (BP) than oral dosing (Soppi et al., 2007; Vinge et al., 1986); however, the nimodipine for injection approved in Europe and other regulated markets contains considerable amounts of organic solvent (23.7% ethanol and 17% polyethylene glycol 400) necessitating serial dilutions and reconstitutions in order to administer the product appropriately to patients at each dosing interval. Further, the amount of alcohol in the currently formulated ex-U.S. medicinal product has the potential to alter the effects of other medicines or neurologic assessments.

1.2. GTX-104

Data from enteral administration studies and observations support the rationale for having an IV administration that would serve an as yet unmet need for patients who receive nimodipine for SAH treatment and exhibit impaired enteral metabolism, excessive nausea, and/or decreased level of consciousness. GTX-104 is a new formulation of nimodipine injection for IV infusion developed by Grace Therapeutics as a sterile aqueous solution containing 10 mg of nimodipine, United States Pharmacopeia (USP) in 5 mL of solution (2 mg/mL). An IV formulation may provide a therapy with lower hurdles to use than the European-approved product.

A detailed overview of clinical experience with GTX-104 and nimodipine is provided in the Investigator’s Brochure and summarized below.

1.2.1. Clinical Experience with GTX-104

GTX-104 has been evaluated in a 4-part, single center, randomized, safety dose-escalation and crossover study in healthy male and female subjects designed to assess the pharmacokinetics (PK), bioavailability (BA), and safety of GTX-104 administered via IV infusion compared to nimodipine oral capsules (Study GTX-104-001). Part 1 evaluated incremental dose-escalation (“titration”) of GTX-104 administered at dose levels of 0.3 mg/h to 1.22 mg/h over 16 hours, with dose-escalation occurring every 4 hours (0.3, 0.6, 0.9, and 1.22 mg/h).

Parts 2, 3, and 4 were two-period, crossover, relative BA studies. Part 2 was a pilot study that evaluated GTX-104 administered open-label as 1.22 mg/h continuous IV infusion for 16 hours compared to oral nimodipine (60 mg every 4 hours [q4h] for 12 hours) in 12 subjects. Part 3 was a BA study, with GTX-104 administered as 1.1 mg/h continuous IV infusion for 28 hours compared to oral nimodipine capsules administered q4h for 24 hours at a dose level of 60 mg in approximately 32 subjects. Part 4 was an extension study and had the same study design as Part 3, only GTX-104 was administered open-label as a continuous IV infusion of 1.4 mg/h for 36 hours with oral nimodipine administered for 20 hours (approximately 24 subjects).

There were no serious adverse events (AEs) in Parts 2, 3, and 4 of the study. In Part 2, 3 (20.0%) subjects reported AEs following administration of IV GTX-104 and 8 (50.0%) subjects reported AEs following administration of oral nimodipine. One subject (Subject 0762) had two clinically significant AEs of atrioventricular block first degree based on electrocardiogram (ECG) findings. In Part 3, 14 (34.1%) subjects reported AEs following administration of IV GTX-104 and 18 (43.9%) subjects reported AEs following administration of oral nimodipine. One subject (Subject 0797) had a clinically significant finding of xeroderma on physical examination. In Part 4, 10 (41.7%) subjects reported AEs following administration of IV GTX-104 and 8 (36.4%) subjects reported AEs following administration of oral nimodipine. One subject (Subject 0983) was withdrawn from the study due to an AE (IV infiltrate).

1.2.2. Clinical Experience with Nimodipine

1.2.2.1. Nimodipine Capsules

The approval of NIMOTOP capsules in 1988 was based on 4 randomized, placebo-controlled trials. The efficacy of orally administered nimodipine in the treatment of patients with SAH

was evaluated in adequate and well-controlled studies of nimodipine oral capsules (NIMOTOP) and nimodipine tablets (NIMOTOP) in patients with SAH. Nimodipine was shown in all four randomized, double-blind, placebo-controlled trials to reduce the severity of neurological deficits resulting from vasospasm in patients who have had a recent SAH (NIMOTOP (nimodipine) capsules for oral use [Approved Label], 2005; NYMALIZE (nimodipine) oral solution [Prescribing Information], 2013), irrespective of the formulation evaluated.

AEs were reported in approximately 11.2% of patients with SAH treated with NIMOTOP; the most frequently reported AE was decreased BP (4.4%). Other AEs that occurred in greater than 1% of the treated population for at least one dose level included cardiac abnormalities (ECG abnormalities, tachycardia, bradycardia) in 0.6 to 1.4% of patients, nausea (0.6% to 1.4%), diarrhea (1.7% to 3.4%), gastrointestinal symptoms (1.2% to 2.4%), edema (0.4% to 1.2%), abnormal liver function tests (0.4% to 1.2%), rash (0.6% to 2.4%), dyspnea (1.2%), muscle pain/cramps (0.2% to 1.4%), and acne and depression (both 1.4%) (NIMOTOP (nimodipine) capsules for oral use [Approved Label], 2005).

1.2.2.2. Intravenous Nimodipine Infusion

The therapeutic efficacy of nimodipine has been evaluated in over 500 patients through a wealth of European clinical studies dating back to 1984, utilizing continuous IV infusion of doses equating to 2 mg/h and 3 mg/h. Overall, IV nimodipine has been given to more than 8,200 patients worldwide. The most frequent AE reported in SAH patients was a decrease in BP. Uncommon (incidence between 1 in 100 and 1 in 1,000) adverse drug reactions (ADRs) include rash, headache, tachycardia, hypotension, vasodilation, nausea, thrombocytopenia, and allergic reaction, and rare (incidence between 1 in 1,000 and 1 in 10,000) ADRs include bradycardia, ileus, transient increase in liver enzymes, injection and infusion site reactions, and infusion site phlebitis (NIMOTOP (0.02% Solution for Infusion) [Summary of Product Characteristics], July 2016).

1.2.3. Potential Benefits and Assessment of Potential Risks

GTX-104 has not been approved for any indication. Efficacy is not being measured in this study, which is being conducted in healthy volunteers; thus, no direct benefits are anticipated. The performance of physical and laboratory evaluations may provide an indirect benefit.

The data from this study will provide important information regarding the relative BA of GTX-104 in the proposed regimen as compared to oral nimodipine. The potential risks are expected to be comparable to those noted in clinical experience with GTX-104 and oral nimodipine (see Section 1.2.1, 1.2.2, and the Investigator’s Brochure) and are considered acceptable in the light of the necessary PK and clinical data to be collected. Population PK (popPK) modeling has been conducted (see Section 10.2), and interim PK data analysis may be conducted (see Section 10.5.1), to ensure an appropriate minimum number of subjects will be enrolled.

1.3. Rationale and Dose Justification

This study is designed to evaluate the relative BA and safety of an IV regimen of GTX-104 as compared to oral nimodipine. The oral dose of 60 mg nimodipine administered q4h was selected because this is the recommended dosage and administration regimen in the most recent labeling (Nimodipine Capsules [Drug Label Information], August, 2012) for

nimodipine capsules, approved and marketed in the U.S.; thus, an adequate safety profile for nimodipine at this oral dose has already been demonstrated.

The dose for GTX-104 was selected based on popPK modeling conducted on data obtained from Parts 2, 3, and 4 of Study GTX-104-001.

A popPK analysis of nimodipine following oral and IV infusion was performed for data from Study GTX-104-001 Parts 2, 3, and 4 (N= 79 subjects, crossover design) using nonlinear mixed effects modeling (NONMEM; version 7.3) and used to derive the test drug regimen. The popPK model was evaluated for parameter precision, and model fitting was evaluated with diagnostic plots and visual predictive checks (VPCs). The relative standard errors (RSE) of the model parameters were mostly within 10%, the diagnostic plots of the popPK model showed unbiased model fitting, and the VPC plots showed consistent concentration-time profiles between the observations and the model simulations for both oral and IV doses.

Following the establishment of the popPK model, simulations were performed to derive a GTX-104 regimen that results in maximum concentration (C_{max}) on Day 1 first dose, and area under the concentration-time curve (AUC) at steady state ($AUC_{Day\ 3,\ 0\text{-}24}$) comparable to those which occur with 60 mg nimodipine oral capsules administered q4h (Figure 1-1, Figure 1-2 and Table 1-1). Additionally, the C_{max} of Day 3 were also comparable for the two treatments (Figure 1-3), with geometric mean differences <10% (Table 1-1). All simulated IV doses commenced at 8 AM. The 4 AM and 8 AM oral doses were given fasted, while the other doses were given in the fed state according to the experience in the GTX-104-001 trial. A total of 1000 patients were used for simulations. The summary of simulated PK endpoints is given in Table 1-1.

Note that for Figure 1-2, as in Figure 1-1, the C_{max} depicted here for the oral regimen does not correspond to the calculated C_{max}, as the time to C_{max} (T_{max}) varies considerably between subjects for the oral dose (due to between subject variability in Ka), but T_{max} is always exactly 0.5 hours for the IV formulation. Also of note is the reduced variability with the IV regimen due to the elimination of variability in BA and food effect, resulting in fewer concentrations greater than 100 ng/mL).

Based on the results of the modeling and simulations, the GTX-104 regimen most likely to be comparable to the NIMOTOP regimen was determined as a 30-minute infusion of 4 mg q4h and a continuous infusion of 0.15 mg/h such that the total drug infused over the first 30 minutes q4h equals 4.075 mg and the total drug infused every 4 hours equals 4.6 mg. This regimen is likely to match $C_{max,Day\ 1\ 8\ AM\ dose,}$ and $AUC_{Day\ 3,\ 0\text{-}24hr}$ for the reference formulation and regimen (NIMOTOP, 60 mg q4h) and result in similar geometric mean C_{max} for Day 3 (all six doses) for both regimens.

Table 1-1. Simulated PK Parameters for Nimodipine Administered as IV and Oral Doses

PK Parameters	Geometric mean (CV)	
	**GTX-104 IV**	**Oral Nimodipine**
$AUC_{Day\ 3\ 0\text{-}24hr}$, ng*h/ml	591 (18.3%)	584 (22.7%)
C_{max} Day 1 (8 AM dose), ng/ml	68.1 (24.0%)	66.6 (51.2%)
C_{max} Day 3 (all doses), ng/mL	88.6 (18.9%)	96.0 (36.5%)

CV = coefficient of variation; IV = intravenous; PK = pharmacokinetics

Figure 1-1. Simulated Concentration-Time Profiles for IV and Oral Regimens on Day 1, First Dose at 8 AM.

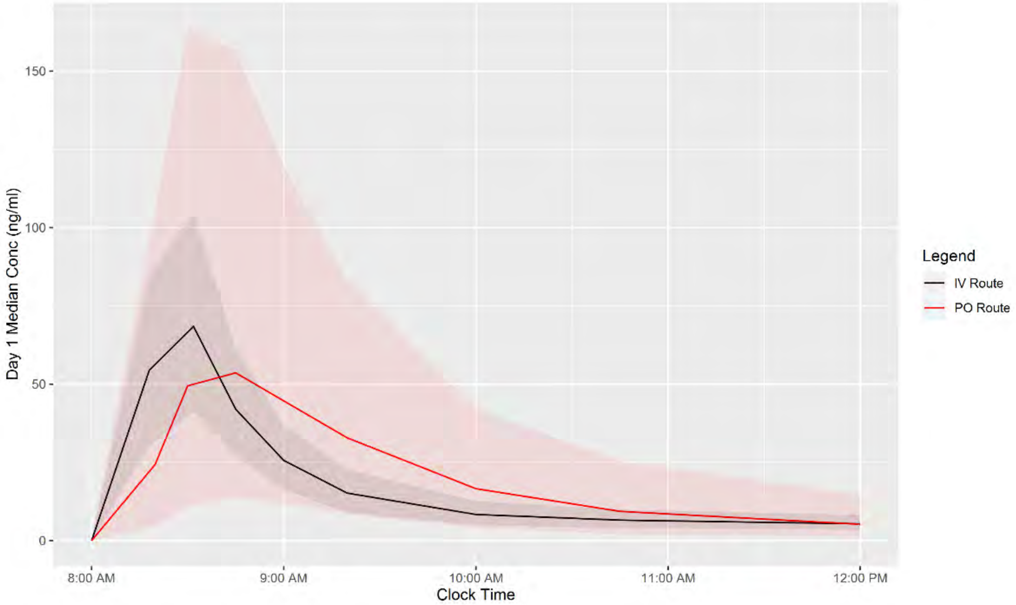

The black line with grey shaded area is the simulated median and 95% CI concentration-time profile for 4 mg over 30 minutes q4h in conjunction with a 0.15 mg/h infusion on Day 1, for the 1st dose. Red line with red shaded area is simulated median and 95% CI concentration-time profile for 60 mg oral route q4h on Day 1, for the 1st dose.

Figure 1-2. Simulated Concentration-Time Profiles for IV and Oral Regimens on Day 3

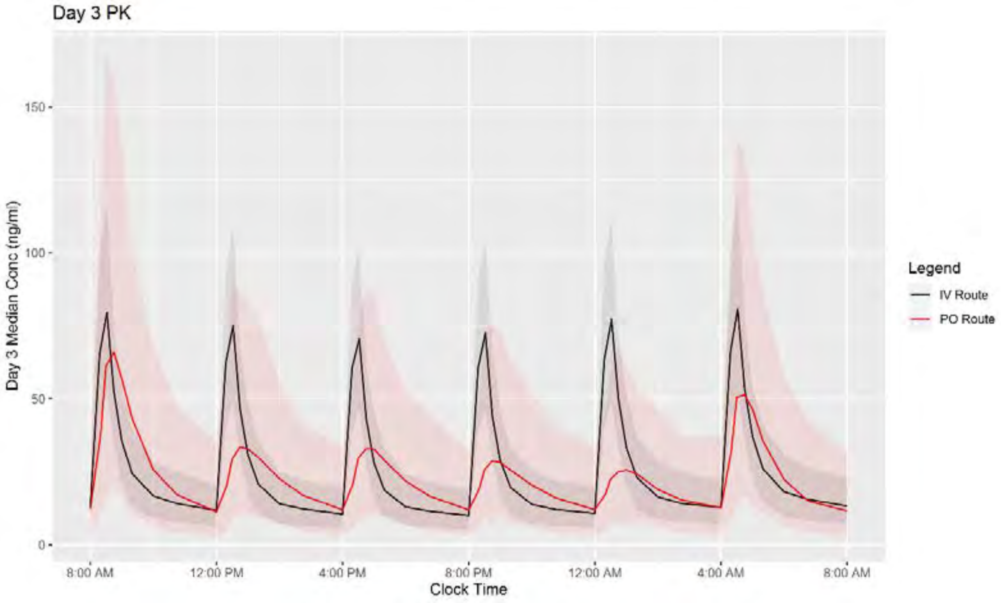

The black line with grey shaded area is the simulated median and 95% CI concentration-time profile for 4 mg over 30 minutes q4h in conjunction with a 0.15 mg/h infusion on Day 3 for all 6 doses. Red line with red shaded area is simulated median and 95% CI concentration-time profile for 60 mg oral route q4h on Day 3 for all 6

doses. Note that the C_{max} depicted above for the oral regimen does not correspond to the calculated C_{max}, as T_{max} varies considerably between subjects for the oral dose (due to between subject variability in absorption rate, Ka), but T_{max} is always exactly 0.5 hours post 4 mg infusion for the IV formulation. The C_{max} for oral dosing, calculated by standard noncompartmental methods, corresponds well to that predicted for the IV regimen.

Figure 1-3. Overall Simulated Cmax on Day 3 for IV and Oral Regimens

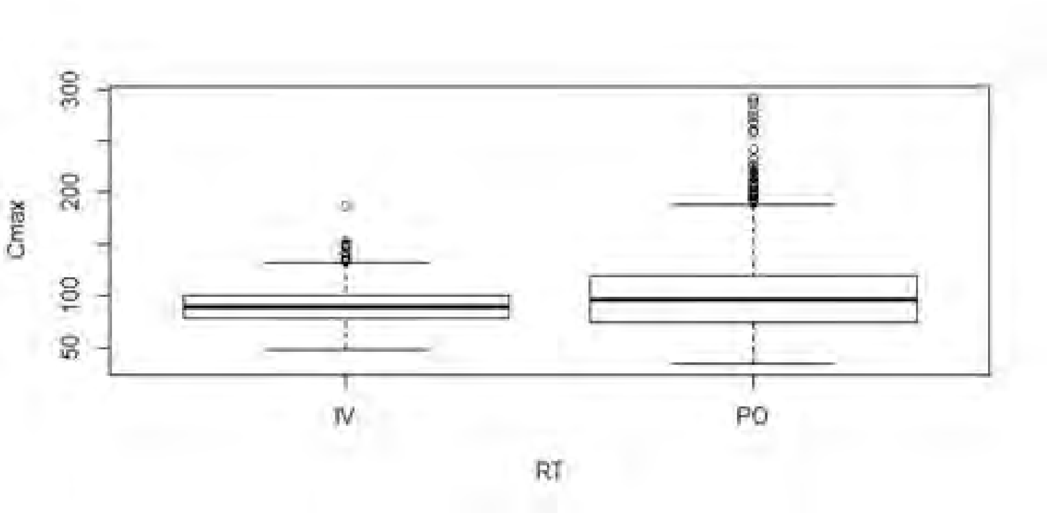

Median, 1st and 3rd quantiles were presented in the box plot for C_{max} values on Day 3 for IV and oral regimens.

1.3.1. Safety Experience with Selected Dose Regimens

There is extensive clinical experience with oral NIMOTOP at 60 mg q4h, including higher administered doses of IV nimodipine than proposed in the current study. In total, the administration of IV nimodipine has been reported in 8,203 patients outside of the US. While IV nimodipine was predominantly administered according to the manufacturer's instructions and local prescribing information (i.e., a dose of approximately 1 mg/h [~15 µg/kg body weight/h] infused continuously for the first 2 hours, increasing as tolerated to 2 mg/h [~30 µg/kg body weight/h] for 5 to 14 days).

Altogether in the literature, 8 reports and 2 abstracts report the administration of IV nimodipine at doses ≥4 mg/h in 260 patients (Adnet et al., 1989; Auer et al., 1983; Boldt et al., 1987; Dalal et al., 1995; Gelmers, 1982; Gomis et al., 2010; Ma et al., 2009; Muller et al., 1987; Wanner-Olsen et al., 2000; Zhang et al., 1995); the 2 abstracts did not detail the AEs and are not discussed here. In the largest study, a prospective double-blind, placebo-controlled, randomized trial (Gomis et al., 2010), 95 patients with aSAH received IV nimodipine at a dose of 5 to 10 mg/h according to BP tolerance followed by oral nimodipine every 4 hours for 3 weeks, which was the maximum duration of administration. The maximum dose administered in any study was 8 mg nimodipine by IV injection over 30 minutes, three times a day (TID) for 14 days in 32 patients with aSAH (Ma et al., 2009). In a prospective study in patients with ischemic stroke, IV administration over ≤10 minutes of 15 µg/kg or 30 µg/kg nimodipine was administered to 10 patients (Gelmers, 1982).

There were no safety concerns identified in 4 of the 8 literature reports (Auer et al., 1983; Dalal et al., 1995; Gomis et al., 2010; Wanner-Olsen et al., 2000). In the study published by Ma et al., 2009 in 32 patients with aSAH, one patient administered IV nimodipine experienced light palpitations or shortness of breath. No serious adverse events (SAEs) were observed in this study (Ma et al., 2009). In a prospective study in 10 patients with acute ischemic stroke, one patient (66-year-old male) without any cardiac history developed a fall in BP and bradycardia 5 minutes after the IV injection of 30 µg/kg nimodipine. BP dropped from 140/80 to 90/60, together with bradycardia (48/min, regular, equal). Within 2.5 minutes the blood pressure was normal with a pulse rate of 92/min (Gelmers, 1982). A study of nimodipine given IV to 10 men with coronary heart disease (1.0 µg/kg/min for 30 min) suggested that nimodipine may dilate pulmonary vessels and increase cardiac output without detrimental effects on intrapulmonary shunting in patients with normal lung function (Boldt et al., 1987).

In the study by Zhang et al., 1995, which examined controlled hypotension induced by nimodipine in craniocerebral surgery (infusion rate of 600 to 800 µg/kg over 30 to 60 minutes; total dose 4 to 12 mg), the extent of decreased blood pressure was 33.0 to 37.7% of mean arterial pressure (MAP), 31.6 to 35.6% of systolic pressure, and 36.1 to 41.9% of diastolic pressure (Zhang et al., 1995). Heart rate was stable in all patients during controlled hypotension, and there were no increases in myocardial oxygen consumption or myocardial ischemia. The authors found it easy to control the level of hypotension. There was no significant fluctuation of BP. Systolic-diastolic BP difference was increased during controlled hypotension. There was no obvious myocardial depression by nimodipine. There was no rebound hypertension after discontinuation of nimodipine, and BP returned to normal levels 15 to 30 minutes after discontinuation.

No deaths or other SAEs were reported in any of the literature reports. There were no other cases of hypotension/decreased blood pressure/low blood pressure or reduced/low mean arterial pressure reported. With the exception of light palpitations and sinus bradycardia of short duration, there were also no cardiac events reported.

1.3.2. Dose Justification

Based on popPK modeling and simulations, the GTX-104 IV dose regimen of a 4 mg dose administered over 30 minutes q4h in conjunction with 0.15 mg/h continuous infusion is likely to match C_{max} on Day 1 and $AUC_{Day\ 3\ 0\text{-}24hr}$ for oral nimodipine q4h and result in similar C_{max} on Day 3 for both IV and oral regimens. Continuous infusions with GTX-104 at rates up to 1.4 mg/h for a day or more were demonstrated to be well tolerated in Study GTX-104-001. A literature review of studies with IV nimodipine administered at doses of up to 8 mg over 30 minutes suggest that the proposed 4 mg dose administered over 30 minutes does not represent a substantial clinical safety risk. Hypotension, as with all nimodipine treatments, is a potential concern; however, several factors facilitate managing the risk of hypotension with the IV regimen. These include:

1. The PK variability from the IV formulation is less than that from the oral regimen (Figure 1-2).
2. The distribution half-life of nimodipine is short (1 to 2 hours). As such, the plasma concentrations are expected to fall rapidly if hypotension required the infusion to be stopped. No option exists for stopping absorption following oral administration.

Overall, the proposed GTX-104 dosing regimen is anticipated to represent acceptable clinical safety and considered likely to match C_{max} and AUC for oral nimodipine q4h. Scheduled BP monitoring is included in the study to closely follow hypotension as a potential AE. Withdrawal criteria for subjects in the event of hypotensive events are described in Section 9.9.

2. STUDY OBJECTIVES

2.1. Primary Objective

The primary objective of this study is to evaluate the relative BA of GTX-104 administered by IV infusion versus nimodipine oral capsules at steady state.

2.2. Secondary Objective

The secondary objective of the study is to assess the safety and tolerability of GTX-104 and nimodipine oral capsules in healthy subjects.

3. STUDY ENDPOINTS

3.1. Primary Endpoints

The following nimodipine PK parameters will be the primary endpoints:

- Maximum concentration (Cmax) on Day 1 for the first dose (8 AM dose)
- Area under the concentration-time curve (AUC) on Day 3 (AUCDay 3, 0-24hr) from 8:00 AM to 8:00 AM

3.2. Secondary Endpoints

Additional nimodipine PK parameters include:

- C_{max} on Day 3 across all 6 doses
- Absolute bioavailability (F)
- Total body clearance of the drug from plasma after IV infusion (CL)
- Apparent total clearance of the drug from plasma after oral administration (CL/F)

3.3. Safety Endpoints

Safety endpoints will include:

- Incidence of treatment-emergent AEs (TEAEs) and SAEs, grouped by system organ class, preferred term, relationship to study drug, severity, and treatment regimen
- Change from baseline in clinical laboratory evaluations (hematology, clinical chemistry, and urinalysis), vital signs, physical examination results, and 12-lead safety ECG parameters at the end of the study

4. INVESTIGATIONAL PLAN

4.1. Overall Study Design

This is a Phase 1, single center, randomized, two-period crossover study in healthy male and female subjects designed to evaluate the relative BA and safety at steady state of two

formulations of nimodipine: GTX-104 (nimodipine for IV infusion; test formulation) and nimodipine oral capsules, RS (reference formulation).

Overall, at least 60 subjects are planned for enrollment in the study in order to have 50 completed subjects. An interim PK data analysis may be conducted to analyze the PK of nimodipine after completion of 20 subjects, as described in Section 10.5.1.

After a 45-day Screening period where eligibility will be assessed, healthy male and female subjects will be admitted to the clinical research unit (CRU) on the day prior to dosing (Day -1) and remain domiciled in the CRU for the duration of the study. At admission, subjects will be randomly assigned in a 1:1 ratio to 1 of 2 treatment sequences: AB or BA, where Treatment A and Treatment B are as follows:

- **Treatment A (GTX-104, Test)**: Nimodipine will be administered by infusion over 72 hours. Administration will be a 30-minute infusion of 4 mg q4h in addition to the continuous infusion of 0.15 mg/h over 72 hours, such that the total drug infused over 30 min every 4 hours will equal 4.075 mg, and the total drug infused every 4 hours will be 4.6 mg.
- **Treatment B (NIMOTOP, RLD)**: Nimodipine capsules (RS) administered orally with 240 mL of water at a dose level of 60 mg (two 30 mg capsules) q4h for 72 hours.

Starting on Day 1, subjects will receive both treatments in the order dictated by their assigned sequence with each treatment period separated by a minimum of 96 hours to allow for treatment washout.

On Days 1 to 4 of each treatment period, study drug will be administered at the same time each day (starting at approximately 8:00 AM on Day 1). Each morning (4:00 AM and 8:00 AM) doses will be administered in fasted status, and breakfast will be provided 2 hours after the 8 AM dose (e.g., subjects should be fasted from 2:00 AM to 10:00 AM). Standardized meals will then be provided at approximately uniform times throughout the day between 10:00 AM to 2:00 AM of the next day, such that the 4:00 AM, and 8:00 AM doses (e.g., the oral dose or the 4.0 mg infusion for GTX-104) are administered to subjects in the fasted state and other doses of 12 PM, 4 PM, 8 PM and 12 AM will be administered in fed status each day. On Days 3 and 10 only, water will be restricted 30 minutes before and 1 hour after administration of both IV and oral doses. Outside of these windows, water will be allowed ad libitum. Detailed information on food and fluid intake are described in Section 6.3.1.

Blood samples for analysis of nimodipine PK will be taken at the timepoints specified in the Time and Events Schedule (Table 4-1) and in Section 8.11. Safety assessments will be collected throughout the study and will include concomitant medications, AEs and SAEs, clinical laboratory evaluations, and resting vital signs (including BP measurements. During Treatments A and B, subjects will undergo BP recording with an oscillometric sphygmomanometer prior to first dose (in each treatment arm) and 60 minutes after dosing for the 8:00 AM, 12:00 PM, 4:00 PM, and 8:00 PM doses on Days 3 and 10. (see the Time and Events Schedule, Table 4-1).

After the final PK sample of the second dosing period, subjects will undergo End of Study (EOS) assessments and be discharged from the CRU, if safety parameters are acceptable to the Investigator.

The study schematic is provided in Section 4.1.1 and the Study Time and Events Schedule is given in Section 4.1.2.

4.1.1. Study Schematic

The study schematic for the full study is provided in Figure 4-1.

Figure 4-1. Study Schematic

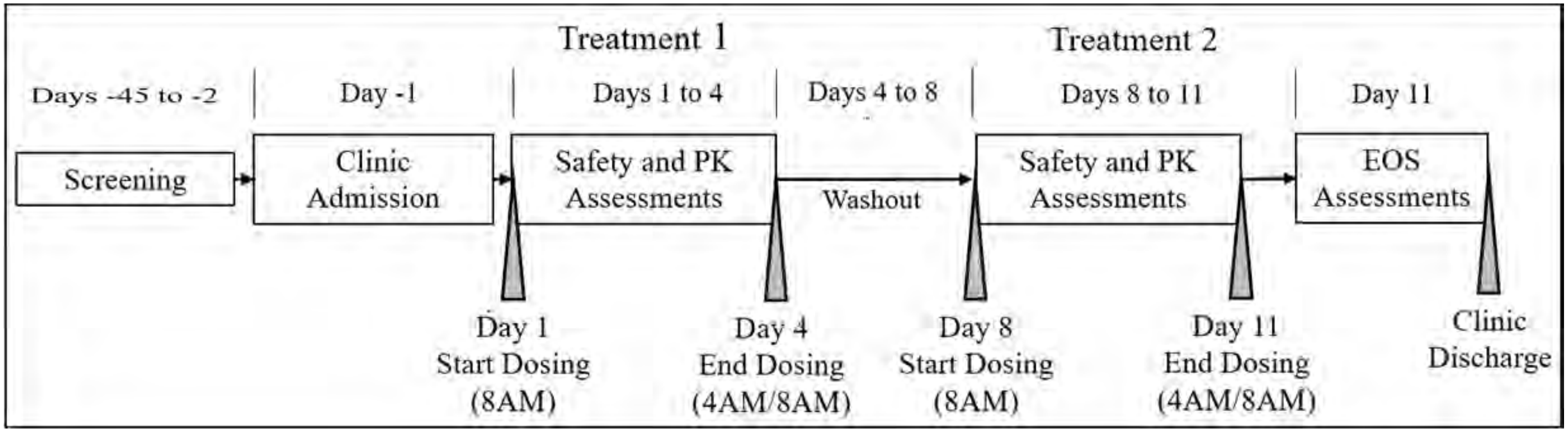

EOS = End of Study; PK = pharmacokinetic

Subjects will be admitted to the clinical research unit (CRU) and randomized to a treatment sequence on Day -1. For treatment sequence AB, Treatment 1 will be Treatment A and Treatment 2 will be Treatment B; for treatment sequence BA, Treatment 1 will be Treatment B and Treatment 2 will be Treatment A. Dosing schematics for Treatment A (GTX-104 IV administration) and Treatment B (oral nimodipine) are provided in Figure 4-2 and Figure 4-3, respectively. In each dosing period, study drug administration will begin at approximately 8 AM in fasted status. The 72-hour dosing period will end on Day 4 for the first treatment and Day 11 for the second treatment, at 4 AM (oral nimodipine) or 8 AM (GTX-104). The washout period will be at least 96 hours. EOS assessments will begin after the final PK assessments of the second treatment period. A detailed study assessment schedule is provided in Table 4-1.

Note that the overall study days provided in the figure assume the minimum washout period of 96 hours. The washout period may be extended as long as study drug initiation occurs at the same time of day (approximately 8 AM). If the washout period is extended, the overall numerical study day for Treatment 2 and the EOS assessments will be different than those presented above.

Nimodipine will be administered based on each subject's randomized treatment schedule: AB or BA, where Treatment A is GTX-104 administered as a 72-hour continuous infusion at 0.15 mg/h in addition to 4 mg administered over 30 minutes q4h (total drug infused over 30 minutes every 4 hours = 4.075 mg, and total drug infused every 4 hours = 4.6 mg), and Treatment B is 60 mg oral nimodipine administered q4h for 72 hours (18 total doses).

The schematic outline for GTX-104 administration (Treatment A) is given in Figure 4-2. For GTX-104 infusion, the 4 mg dose will be administered at the same time as the start of the continuous infusion on Day 1 of the dosing period. The end of the continuous 0.15 mg/h infusion will be 8 AM on Day 4 of the study dosing period. Note that if GTX-104 is given as Treatment 2, and if the washout period is the minimum time, Day 1, 2, 3, and 4 of the dosing period will correspond to overall Study Days 8, 9, 10, and 11. The washout period may be extended as long as study drug initiation occurs at the same time of day (approximately 8 AM). If the washout period is extended, the overall numerical study day for Treatment 2 and the EOS assessments will be different than the aforementioned days.

The beginning of infusion on Day 1 should be administered after at least a 10-hour overnight fast, with breakfast provided 2 hours after the 8:00 AM dose (i.e., subjects fasted from 10:00 PM the night before to 10:00 AM). On Days 2-4, subjects need to be fasted between 2:00 AM-10:00 AM every day. Standardized meals will then be provided at approximately uniform times throughout the day, as described in Section 6.3. PK and clinical venous blood sampling should occur in the arm opposite to infusion, and the infusion line should not be used for PK sampling. For both Treatment A and Treatment B, oscillometric sphygmomanometer BP monitoring should occur prior to the first dose and 60 minutes post dose for the 8:00 AM, 12:00 PM, 4:00 PM, and 8:00 PM doses on Days 3 and 10, as specified in Table 4-1 and described in Section 8.10.

If Treatment A is given as Treatment 2, EOS assessments should be completed as described in Table 4-1 after the final PK blood sample collection.

Figure 4-2. Study Drug Administration Schematic (Treatment A; GTX-104)

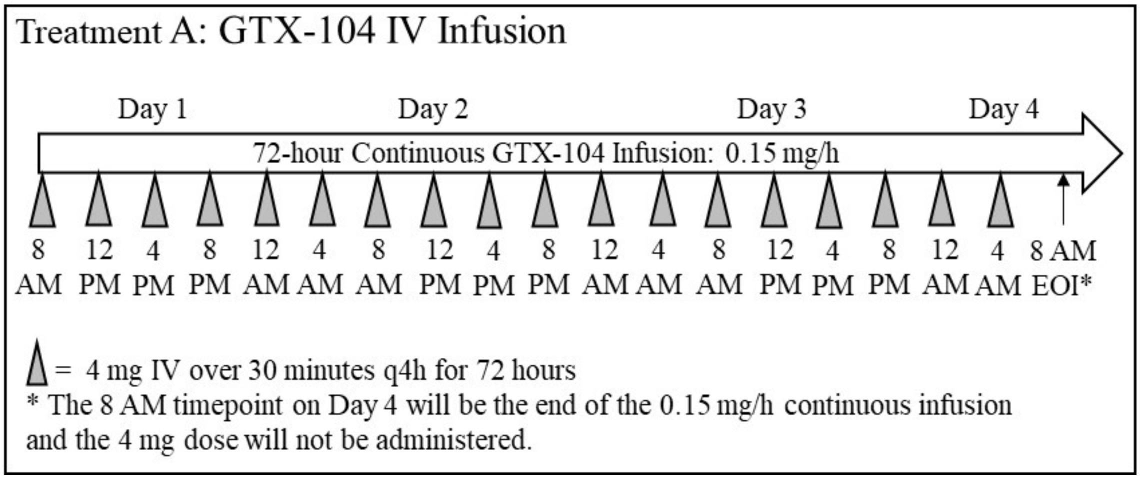

EOI = end of infusion; IV = intravenous; q4h = every 4 hours

Note: In the event that Treatment A is given as the 2nd treatment after the minimum washout period, Day 1, 2, 3, and 4 will correspond to overall Study Days 8, 9, 10, and 11, respectively. The overall study days should be

adjusted if the washout period is longer. Note that this assumes the minimum washout period. The washout period may be extended as long as study drug initiation occurs at the same time of day (approximately 8 AM). If the washout period is extended, the overall numerical study day for Treatment 2 and the EOS assessments will be different than the aforementioned

The schematic outline for oral nimodipine administration (Treatment B) is given in Figure 4-3. Dosing with oral nimodipine should begin at approximately 8:00 AM on Day 1 of the dosing period at a dose level of 60 mg (two 30 mg capsules) q4h, with a total of 18 doses administered over the 72-hour dosing period. Note that if oral nimodipine is given as Treatment 2, and if the washout period is the minimum 96 hours, Day 1, 2, 3, and 4 of the dosing period will correspond to overall Study Days 8, 9, 10, and 11. The overall study days should be adjusted if the washout period is longer. The washout period may be extended as long as study drug initiation occurs at the same time of day (approximately 8:00 AM). If the washout period is extended, the overall numerical study day for Treatment 2 and the EOS assessments will be different than the aforementioned days.

The first dose of Day 1 should be administered after at least a 10-hour overnight fast, with breakfast provided 2 hours after the 8:00 AM dose (e.g., subjects fasted from 10:00 PM the night before to 10:00 AM). On Days 2-4, subjects need to be fasted between 2:00 AM- 10:00 AM every day. Standardized meals will then be provided at approximately uniform times throughout the day, as described in Section 6.3.

If Treatment B is given as Treatment 2, EOS assessments should be completed as described in Table 4-1 after the final PK blood sample collection.

Figure 4-3. Study Drug Administration Schematic (Treatment B; Oral Nimodipine)

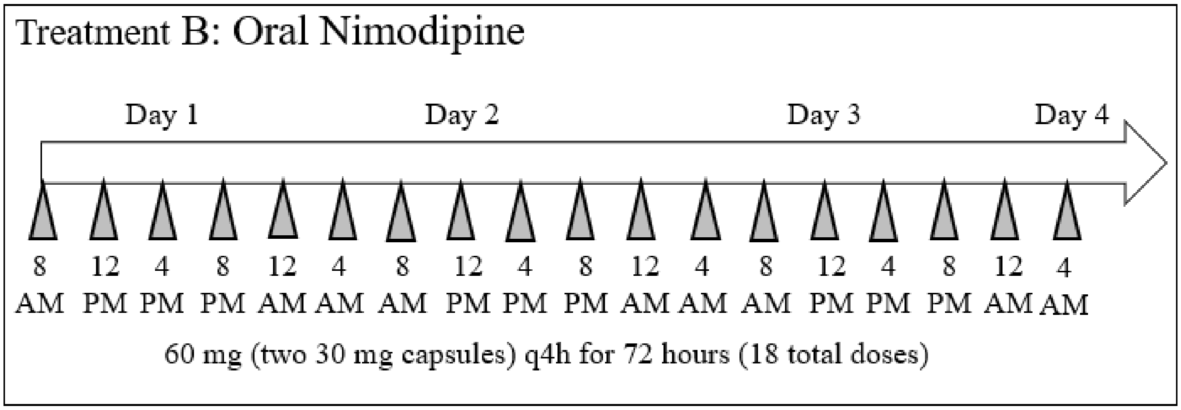

q4h = every 4 hours

4.1.2. Time and Events Schedule

Table 4-1. Time and Events Schedule

**Study Period**	**Screening**	**Admission**	**Treatment**			**End of Study**
			**Treatment 1** [a]	**Washout** [b]	**Treatment 2** [a]	**EOS/ET** [c]
**Overall Study Day**	-45 to -2	-1	1 to 4	4 to 8 [d]	8 to 11 [d]	11 [d]
Informed consent	X					
Subject ID number assignment	X					
Confirmation of eligibility	X	X			X	
Randomization		X				
Medical history & baseline demographics	X	X [e]				
Prior medication assessment	X	X				
Full physical examination	X					
Brief physical examination		X				X
Height measurement	X					
Weight measurement	X	X				X
Body mass index	X					
12-lead electrocardiogram	X					X
Vital signs [g]	X	X	X [f]	X	X [f]	X
Orthostatic hypotension screen	X	X				
Clinical laboratory (chemistry, hematology, coagulation, urinalysis)	X	X				X
Pregnancy test [h]	X	X				
COVID-19 virus test		X[i]		X[j]		
FSH test [k]	X					
Serology [l]	X					
HbA1c	X					
CYP3A4 genotyping	X					
Urine drug and alcohol screen	X	X				
Clinic admission		X				
Study drug administration			X[m]		X[m]	
Standardized meals [n]			X		X	
Pharmacokinetic sampling [o]			X		X	
Adverse events assessments			← Continuous →			
Concomitant medications			← Continuous →			
Clinic discharge						X

CYP = cytochrome P450; EOS = End of Study; ET = Early Termination; FSH = follicle-stimulating hormone; hbA1c = hemoglobin A1c; ID = identification

a. For treatment sequence AB, Treatment 1 will be Treatment A (GTX-104) and Treatment 2 will be Treatment B (oral nimodipine); for treatment sequence BA, Treatment 1 will be Treatment B (oral nimodipine) and Treatment 2 will be Treatment A (GTX-104).
b. Each treatment period should be separated by a minimum of 96 hours to allow for treatment washout; washout time will be different for subjects, depending on their assigned treatment sequence (see Section 6.3).
c. End of Study (EOS) assessments should be attempted in the event of Early Termination (ET). If the subject consents, this should include a blood sample for pharmacokinetic (PK) analysis taken as soon as possible after the decision to withdraw has been made.
d. Overall Study Day may vary depending on the length of the washout period. The washout period should be at least 96 hours and adjusted such that the first dose administration of treatment 2 starts at approximately 8 AM.
e. Confirmation of medical history and update, if needed.
f. On Days 1 and 8 vital signs should be recorded within 1 hour prior to the first dose. On Days 3 and 10, blood pressure should be at 1 hour post-dose (for both IV and oral dose) for the 8:00 AM, 12:00 PM, 4:00 PM 8:00 PM doses. Measurements should be performed as described in Section 8.10. The same arm is to be used for all BP determinations.
g. Unless otherwise noted (i.e., Days 3 and 10, see "f" above), blood pressure measured approximately q12h while subjects are in-clinic or before collection of PK blood samples, as appropriate.
h. Serum pregnancy test (β-human chorionic gonadotropin [β-hCG]) for female subjects of childbearing potential.
i. COVID-19 virus test to be collected within 5 days of CRU admission.
j. COVID-19 virus test to be collected in the morning of Day 4 and on Day 7 on admission, with results to be available by Day 8, start of Period 2.
k. For postmenopausal female subjects.
l. Human immunodeficiency virus (HIV), hepatitis C (HCV), and hepatitis B surface antigen (HBsAg); will be tested at Screening only.
m. Based on subjects' randomized treatment sequence (AB or BA) GTX-104 or oral nimodipine should be administered as described in Section 4.1.1 and Section 6.2. GTX-104 administration will be a 30-minute infusion of 4 mg every 4 hours (q4h) in addition to the continuous infusion of 0.15 mg/h; nimodipine capsules will be administered orally with 240 mL of water at a dose level of 60 mg (two 30 mg capsules) q4h for 72 hours. For Treatment A, the continuous infusion will be completed at 72 hours post-start of infusion (approximately 8 AM on the fourth day of the treatment period); no 4 mg dose will be given at 72 hours.
n. Meals and water should be provided during dosing as described in Section 6.3.1.
o. Blood samples for PK analysis should be taken based on the treatment. For GTX-104 (Treatment A), samples should be taken on the first day of Treatment A, Pre-dose (time 0, before the 1st [8 AM] dose), and at 0.33, 0.5, 0.75, 1, 1.33, 2, 3, and 4 hours post initial (8 AM) dose, and starting on the third day at 48 (prior to initiation of the 4 mg dose), 48.33, 48.5, 48.75, 49, 49.33, 50, 51, 52, 52.33, 52.5, 52.75, 53, 53.33, 54, 55, 56, 56.33, 56.5, 56.75, 57, 57.33, 58, 59, 60, 60.33, 60.5, 60.75, 61, 61.33, 62, 63, 64, 64.33, 64.5, 64.75, 65, 65.33, 66, 67, 68, 68.33, 68.5, 68.75, 69, 69.33, 70, 71, 72-hours post-start of 1st infusion. PK and clinical venous blood sampling should occur in the arm opposite to infusion, and the infusion line should not be used for PK sampling. Note, for timepoints that are scheduled at the same time as the 4 mg dose (0, 48, 52, 56, 60, 64, 68, and 72 hours post-start of infusion), blood for PK analysis should be taken before initiation of the 30-minute infusion. For the 0.5, 48.5, 52.5, 56.5, 60.5, 64.5, and 68.5-hour timepoints, blood samples for PK analysis should be collected after completion of the 4 mg infusion. For oral nimodipine (Treatment B), PK samples taken for the 72-hour oral dosing period, listed sequentially, samples should be taken on the first day of Treatment B: Pre-dose (time 0, before the first [8 AM] dose), and at 0.33, 0.5, 0.75, 1, 1.33, 2, 3, and 4 hours post-dose, and on the third day blood samples for PK should be taken at: 48 (prior to initiation of the 8 AM 60 mg dose), 48.33, 48.5, 48.75, 49, 49.33, 50, 51, 52, 52.33, 52.5, 52.75, 53, 53.33, 54, 55, 56, 56.33, 56.5, 56.75, 57, 57.33, 58, 59, 60, 60.33, 60.5, 60.75, 61, 61.33, 62, 63, 64, 64.33, 64.5, 64.75, 65, 65.33, 66, 67, 68, 68.33, 68.5, 68.75, 69, 69.33, 70, 71, 72-hours since the first dose. Note, for timepoints that are scheduled at the same time as the 60 mg oral dose (48, 52, 56, 60, 64, 68, and 72 hours since first dose), blood for PK analysis should be taken before oral dose intake. See Section 8.11 for PK sampling schedule separated by Day and treatment.

   In the event of ET, a blood sample for PK analysis should be taken as soon as possible after the decision to withdraw, if the subject consents.

4.2. Scientific Rationale for Study Design

This is a standard crossover study designed to evaluate the relative BA at steady state of two formulations of nimodipine: GTX-104 (nimodipine for IV infusion; test formulation) and nimodipine oral capsules, RS (reference formulation). A crossover design was selected to reduce the impact of inter-subject variability in PK analysis. The design is in accordance with the most current guidance from the U.S. Food and Drug Administration (FDA), FDA Bioavailability Studies Submitted in NDAs or INDs — General Considerations: FDA Guidance for Industry, 2019.

The washout period of at least 96 hours was selected based on the half-life of nimodipine to limit the possibility of study drug carryover effects.

4.3. Study Procedures

Note: Written informed consent must be obtained prior to performing any study-related procedure, including subject identification (SID) number assignment and Screening evaluations.

4.3.1. Screening (Study Day -45 to Day -2)

All study candidates will be screened to assess eligibility for enrollment into the study. A Screening Log will be maintained for all consented study candidates. Screen Failures, as defined in Section 5.3, will have the study candidate's unique SID (assigned as described in Section 8.2), date screened, and reason(s) for screening failure captured by electronic data capture (EDC).

During Screening, procedures and assessments will be performed and recorded as detailed in the Time and Events Schedule (Table 4-1).

COVID-19 virus testing will be performed on all study candidates after Day -5.

4.3.2. CRU Admission (Day -1)

Subjects will be admitted to the CRU on the day prior to dosing. Eligibility will be reconfirmed as described in Table 4-1. Subjects will be randomized at admission, as described in Section 6.4. Any AEs that occur prior to study drug administration will be recorded as medical history. Thermometer reading will be done on Day -1. Any subject whose temperature is elevated (>100.2°F; 37.9°C) will not be admitted to the CRU.

4.3.3. Treatment Period (Day 1 to 11)

4.3.3.1. Study Drug Administration (Days 1 to 11)

Note: The overall study days provided assume the minimum washout period, which must be at least 96 hours. However, the washout period may be extended as long as study drug initiation occurs at the same time of day (approximately 8 AM). A washout of 96 hours must occur even if the Day 1 must be repeated, as described in Table 8-2. If the washout period is extended beyond the minimum, the overall numerical study day for Treatment 2 and the EOS assessments will be different than those described in this section.

The treatment period will begin at first study drug administration on Day 1. The treatment period for each subject will be comprised of two 72-hour treatment periods, separated by a minimum 96-hour washout, with study drug administered according to the subject's

treatment sequence (AB or BA). The treatment period will be completed at final CRU discharge (approximately Day 11).

All study procedures will be conducted as described in the Time and Events Table (Table 4-1). On Days 1 to 4 of each treatment period (Days 1 to 4 for the first treatment and approximately Days 8 to 11 for the second treatment), study drug will be administered at the same time each day (starting at approximately 8 AM on Day 1). Each morning (4 AM and 8 AM) doses will be administered in fasted status, and breakfast will be provided 2 hours after the 8 AM dose (e.g., subjects should be fasted from 2 AM to 10 A M). On Day 1, since the first dose is at 8 AM, subjects can be fasted overnight and get the first breakfast at 10 AM, 2 hours after the first dose.

Standardized meals will then be provided at approximately uniform times throughout the day, such that the 4 AM, and 8 AM doses (e.g., the oral dose or the 4 mg infusion for GTX-104) for each treatment are administered to subjects in the fasted state, and other doses (12 PM, 4 PM, 8 PM and 12 AM) were given in fed status each day. On Days 3 and 10 only, water will be restricted 30 minutes before and 1 hour after administration of both IV and oral doses. Outside of these windows, water will be allowed ad libitum. Water will be administered with study drug, and 1 hour before and after initiation of infusion for Treatment A. Outside of this window, water will be allowed *ad libitum*.

For both Treatment A and Treatment B, baseline (prior to first dose) BP determination will be taken via oscillometric sphygmomanometer. On Days 3 and 10, 1 hour post dose BP determinations will be performed after the 8:00 AM, 12:00 PM, 4:00 PM and 8:00 PM doses, as described in Section 8.10.

Any new AEs that occur after treatment administration will be attributed to the formulation administered in that dosing period. Any new AEs that occur during washout will be attributed to the treatment administered during the first dosing period.

4.3.4. End of Study/Early Termination (Day 11)

EOS assessments should be completed after the final PK sample collection and prior to subject discharge from the CRU, as described in the Time and Events Schedule (Table 4-1). In the event of Early Termination (ET), EOS assessments should be attempted, if the subject consents. ET assessments should include collection of a blood sample for PK analysis; the PK time point closest to the ET time and the relative dosing day shall be recorded in the appropriate eCRF.

4.4. Discontinuation Criteria for Subjects and Trial

The study may be prematurely terminated at any time by the Sponsor, Institutional Review Board (IRB), and/or the Investigator in the interest of subject safety and welfare. The Sponsor reserves the right to discontinue the study at any time for any reason.

Section 5.3 describes the procedures to be followed if an individual subject withdraws from the study.

4.5. Duration of Treatment

The study will be defined by a Screening Period (45 days), CRU admission, and two 72-hour treatment periods separated by a minimum 96-hour washout period (at least 12 days

domiciled in the CRU). Participant duration in this study, from Screening to EOS assessments, is planned to be approximately 40 days.

4.6. End of Study Definition

The end of the study for each subject is defined as completion of all described assessments, up to and including EOS/ET assessments.

5. STUDY POPULATION

This study will enroll approximately 60 subjects in order to have 50 completed healthy male and female subjects, based on sample size calculations (Section 10.2).

5.1. Inclusion Criteria

Subjects are eligible for inclusion only if all of the following criteria are met:

1. An IRB-approved informed consent form is signed and dated prior to any study-related activities.
2. Subject is 18 to 55 years of age, inclusive, at time of consent.
3. Subject has a body mass index (BMI) between 18 and 32 kg/m^2, inclusive.
4. Negative COVID-19 virus test within 5 days of CRU admission.
5. Subject is able to communicate clearly with the Investigator and study staff, and willing and able to understand and follow instructions, comply with the protocol requirements, and make all required study visits.
6. Subject is in good general physical health as determined by absence of clinically significant medical or psychiatric history, physical examination findings, vital signs, clinical laboratory evaluations, and 12-lead ECG measurements.
    - The 12-lead ECG should be consistent with normal cardiac conduction and function at Screening, including ventricular rate between ≥45 and ≤100 beats per minute (bpm), a corrected QT (QTc) interval ≤450 ms for male subjects or ≤470 ms for female subjects; QRS interval <120 ms; PR interval <220 ms; and morphology consistent with healthy conduction and function. 12-lead ECGs may be repeated once, at the discretion of the Investigator.
7. Subject has vital signs at Screening that are stable (measured in sitting or semi-reclined position after at least 5 minutes of rest) and are within the following ranges:
    - Systolic blood pressure (SBP): ≥100 and ≤150 mmHg
    - Diastolic blood pressure (DBP): ≥55 and ≤100 mmHg
    - Heart rate: ≥45 and ≤100 bpm

    Note: If vital signs are out-of-range, the Investigator may obtain one additional reading so that up to 2 consecutive assessments are made within 1 hour with the subject seated quietly during the 5 minutes preceding the assessment.
8. Subject is a nonsmoker (for at least 6 months) and does not use tobacco-containing products (including but not limited to, cigarettes, pipes, cigars, chewing tobacco, or nicotine patch or gum).

9. Subject has not consumed and agrees to abstain from taking any prescription drugs, (except as authorized by the Investigator **AND** Medical Monitor) for 7 days prior to first dose of study drug and continuing through EOS.
10. Subject has not consumed and agrees to abstain from taking dietary supplements including vitamins and herbal preparations, or non-prescription drugs (except as authorized by the Investigator **AND** Medical Monitor) for 30 days prior to first dose of study drug and continuing through EOS.
11. Subject has clinical chemistry, serology (screening only), hematology, coagulation (fasted), and complete urinalysis results at Screening and admission to the CRU within the reference range for the testing laboratory, unless the out-of-range results are deemed not clinically significant by the Investigator.
12. Subject has a negative urine drug screen at Screening and at CRU admission.
13. Subject is willing to submit a buccal swab or blood test for genetic testing to identify metabolic genotype for CYP3A4.
14. Female subjects only must have a negative pregnancy test (serum β-human chorionic gonadotropin [hCG]) at Screening and at CRU admission and must not be lactating.
15. Male subjects who are nonsterilized and sexually active with a female partner of childbearing potential and female subjects of childbearing potential must agree to use a protocol-approved method of contraception from signing of informed consent, throughout the duration of the study and for 30 days after the last dose of study drug.
16. Female subjects of nonchildbearing potential must be surgically sterile (i.e., hysterectomy, bilateral oophorectomy, or bilateral tubal ligation) and at least 6 months post-surgery, or must be menopausal, defined as no menstrual period for at least 12 months and confirmed by follicle-stimulating hormone (FSH) level of ≥40 IU/L.
17. Male subjects only must agree to not donate sperm during the study and for at least 30 days after their final dose of study drug.
18. Subject has good bilateral venous access, as judged by the Investigator or designee.

5.2. Exclusion Criteria

Subjects are ineligible for the study if any of the following criteria are met:

1. Does not meet all inclusion criteria.
2. Positive test for COVID-19 virus. Subjects will be retested for COVID-19 virus with sample collected on Day 4. If Day 4 test is positive, subject will be discharge from the unit and withdrawn from the study.
3. History or presence of clinically significant medical illness, including, but not limited to, cardiovascular, pulmonary, hematologic, endocrine, immunologic, dermatologic, neurologic, psychiatric, renal, hepatic, chronic respiratory, or gastrointestinal disease, that could interfere with the interpretation of the study.
4. Has current or recent (within 6 months) history of gastrointestinal disease or any surgical or medical condition (e.g., Crohn's or liver disease) that could potentially alter the absorption, metabolism, or excretion of the study drug.

5. Has any medical condition, physical exam finding, out-of-normal-range laboratory value, or 12-lead ECG at Screening or admission that has not been reviewed, approved, and documented as not clinically significant by the Investigator.
6. Prior history of clinically significant abnormal ECG (e.g., second- or third-degree heart block, uncontrolled arrhythmia, QTcF [Fridericia's correction] interval >450 ms for male subjects or >470 ms for female subjects) or abnormal cardiovascular exam at Screening that does not fall within values given for SBP, DBP, and heart rate in the inclusion criteria.
7. Subject has symptomatic bradycardia, symptomatic hypotension, or any prior history of myocardial infarction.
8. Positive test for orthostatic hypotension.
9. History or presence of malignancy within the past 5 years, except for adequately treated localized skin cancer (basal cell or squamous cell carcinoma).
10. Suffers from clinically significant systemic allergic disease or has a history of significant drug allergies, including, but not limited to, a history of anaphylactic reactions, allergic reactions due to any drug leading to significant morbidity, or known hypersensitivity to any compound in the study products or related compounds.
11. Subject has donated >500 mL or more of blood (or had equivalent blood loss) within 3 months prior to drug administration or intends to donate blood (to blood bank) within 3 months after completion of the study.
12. Has had an acute, clinically significant illness within 30 days prior to first study drug administration on Day 1 or has had a recent febrile illness with an abnormal body temperature for at least 72 hours before dosing on Day 1.
13. Has a history (within 12 months before Screening) of illicit drug abuse or has positive test for drugs of abuse at any time.
14. Has a history (within 180 days of Screening) of alcohol abuse, defined as alcohol consumption of more than 2 drinks per day [maximum 14 drinks per week] for males or more than 1 drink per day [maximum 7 drinks per week] for females, and/or has a positive alcohol test at any time during the study, or is unwilling to abstain from alcohol throughout the study. One drink = 5 ounces of wine, 12 ounces of beer, or 1.5 ounces of hard liquor).
15. Has a smoking history during the past 6 months (calculated from first dosing), including the use of any nicotine-containing substances (e.g., nicotine patch or gum, chewing tobacco, e-cigarettes), or has a positive cotinine test at Screening or upon admission to the CRU, or is unwilling to abstain from these products for the duration of the study.
16. Has had treatment with an investigational drug or experimental medical device within 28 days before study drug administration or five half-lives of the study drug's elimination half-life, whichever is longer.
17. Subject is a known CYP3A4 intermediate or poor metabolizer, or screening genotyping indicates subject is a CYP3A4 intermediate or poor metabolizer.
18. Has used any of the following types of medications:

- Received a strong or moderate inhibitor of CYP3A4 within 14 days or 5 half-lives, whichever is longer, of dosing on Day 1.
- Received more than 1 dose of a CYP3A4 inducer within 30 days before admission to the CRU.
- Within 2 weeks before Day 1: Any therapy that is known to interfere with glucuronidation of drugs, or that is known to exacerbate renal dysfunction (e.g., amphotericin B, aminoglycoside antibiotics, trimethoprim, nonsteroidal anti-inflammatory drugs [NSAIDs]), or antihypertensive agents that excessively reduce cardiac output or systemic BP. Over-the-counter medications such as acetaminophen, topical medication, and nutritional and vitamin supplements will be allowed at the discretion of the Investigator.
- Within 30 days before Day 1: Oral contraceptives, hormone replacement therapy, or any estrogen-containing medications, any monoamine oxidase inhibitors (MAOIs), prescription medicines or herbal preparations (e.g., St John's wort), or received any immunizations.
- Within 3 months before Screening: Opioids, neuroleptics, lithium, antidepressants, mood stabilizers, benzodiazepines, cognitive enhancers, centrally acting antihypertensives, or γ-aminobutyric acid (GABA) agonists.
- Within 6 months before Screening: Any implanted or injected testosterone product; subject must also agree to not use testosterone replacement products (including, but not limited to, topical, nasal, sublingual, or oral testosterone products) for the duration of the study through completion of the EOS assessments.
- Has consumed grapefruit, pomelo, or Seville orange-containing foods or beverages within 7 days before admission to the CRU, or has consumed any products containing caffeine and/or xanthine within 72 hours prior to admission, or is unwilling to abstain from these products during the inpatient portion of the study.

19. If female, the subject is pregnant or lactating, or intends to become pregnant before, during, or within 30 days after participating in this study; or intends to donate ova during such time period.
20. If male, the subject intends to impregnate others, or donate sperm during the course of this study or within 30 days after participating in this study.
21. Has inadequate venous access for the required blood draws for the study.
22. Is unable to meet or perform study requirements or has a known or suspected inability to comply with the study protocol.
23. Is unable or unwilling to eat provided food (e.g., vegetarian, kosher, lactose-intolerant).
24. Is an immediate family member of the Investigator, or an employee of the study center, with direct involvement in the proposed study, or other studies under the direction of the Investigator or study center, or is in a dependent relationship with a study center employee who is involved in the conduct of this study (e.g., spouse, parent, child, sibling), or may consent under duress.

5.3. Screen Failures

Screen failures are defined as participants who sign informed consent forms (ICFs) to participate in the clinical trial but are not subsequently randomized due to not meeting inclusion or exclusion criteria. Screen failures will be recorded in a Screening Log that will record the study candidate's unique SID, date screened, and reason(s) for screening failure.

5.4. Removal of Subjects

Subjects will be advised that they are free to withdraw from the study at any time for any reason, without prejudice to their medical care. A subject may be withdrawn from the study for any of the following reasons:

- Subject voluntarily discontinues study participation (subject withdrawal)
- The need to take medication which may interfere with study measurements
- Intolerable/unacceptable AEs
- Hypotensive AEs, with heart rate, SBP, or DBP meeting the criteria as defined in Appendix 2 or at the discretion of the Investigator
- Major violation or deviation of study protocol procedures
- Non-compliance of subject with protocol or inability to comply with study procedures
- Withdrawal from the study, in the Investigator's judgment, is in the subject's best interest
- Subject is lost to follow-up
- Study termination by the Sponsor

The primary reason for withdrawal or discontinuation must be recorded on the subject's electronic case report form (eCRF) and, if necessary, on an AE form if the subject is withdrawn due to an AE. EOS assessments, including a blood sample for PK analysis collected as soon as possible after the decision to withdraw has been made, will be attempted in the event of ET, if the subject consents.

All data gathered prior to withdrawal will be made available to the Sponsor for use in the study report.

5.5. Replacement of Subjects

Subjects who withdraw, or are withdrawn, during Screening will be replaced to ensure that at least 50 subjects are enrolled into the study. Subjects who withdraw during the study may be replaced at the discretion of the Sponsor.

6. TREATMENT OF SUBJECTS

6.1. Description of Treatments

6.1.1. Investigational Product

GTX-104 (nimodipine injection for IV infusion) will be provided as a sterile aqueous light yellow to yellow color solution. Each 5-mL amber glass vial will contain 10 mg of nimodipine USP (2 mg/mL), and inactive ingredients (polysorbate 80 USP, ethyl alcohol 95% USP, and water for injection USP). IV dosing solutions will be prepared on-site by a Pharmacist or designee and will be distributed as required to the study team. Specific details

regarding investigational product supplies, dose preparation, any special considerations for product and dose stability and ancillary materials, and accountability will be provided in the Pharmacy Reference Information document supplied to the study center.

6.1.2. Reference Product

Nimodipine capsules, RS (BION Pharma Inc.) for oral use will be supplied as oblong, white opaque, soft gelatin capsules with NM1 printed in black ink, in unit-dose packages of 30 or 100 capsules, with each capsule containing 30 mg of nimodipine.

6.2. Treatments Administered

Treatment A (Test): Nimodipine will be administered by infusion over 72 hours. Administration will be a 30-minute infusion of 4 mg q4h in addition to the continuous infusion of 0.15 mg/h (total drug infused over 30 min every 4 hours = 4.075 mg; total drug infused q4h = 4.6 mg).

Treatment B (Reference): Nimodipine capsules (RS) administered orally with 240 mL of water at a dose level of 60 mg (two 30 mg capsules) q4h for 72 hours for a total of 18 doses.

Dosing schematics for Treatment A (GTX-104 IV administration) and Treatment B (oral nimodipine, RS) are provided in Figure 4-2 and Figure 4-3, respectively (Section 4.1.1).

6.3. Timing of Dose

On Days 1 to 4 of each treatment period, study drug will be administered at the same time each day (starting at approximately 8 AM on Day 1). The time of dosing will be standardized to avoid effects of diurnal variation between dosing periods.

For GTX-104, the 30-minute 4 mg infusion should be started at the same time in the morning (e.g., 8 AM) as the 0.15 mg/h continuous infusion. The 4 mg infusion should be administered q4h starting on Day 1 (e.g., 8 AM, 12 PM, 4 PM, 8 PM, 12 AM, 4 AM) for a total of 18 infusions of the 4 mg dose. The continuous infusion should be stopped on Day 4 after 72 hours (e.g., approximately 8 AM).

Oral nimodipine will be administered at a dose level of 60 mg (two 30 mg capsules) q4h starting at approximately 8 AM on Day 1 of the dosing period, for a total of 18 doses (e.g., 8 AM, 12 PM, 4 PM, 8 PM, 12 AM, 4 AM).

The time of the washout period will be different for each treatment sequence (AB or BA). For the AB treatment sequence, the washout will be at least 96 hours. However, for the BA treatment sequence, as the final dose of oral nimodipine is given at 4 AM on Day 4, the washout duration will be at least 100 hours. In all cases, washout should meet or exceed a minimum of 96 hours.

6.3.1. Food and Water Intake

The morning (4 AM and 8 AM) doses (e.g., the oral dose or the 4 mg infusion for GTX-104) will be administered in fasted status, and breakfast will be provided 2 hours after the 8 AM dose (e.g., subjects should be fasted from 2 AM to 10 AM).

Standardized meals will then be provided at approximately uniform times throughout the day, such that the 4 AM, and 8 AM doses (the 4 mg infusion for GTX-104) for each treatment are administered to subjects in the fasted state and other doses (12 PM, 4 PM, 8

PM, 12 AM) are administered in fed status. Meals will be approved by the Sponsor and will be administered at approximately the same time each day, as is feasible.

Water will be restricted from 30 minutes before to 1 hour after administration of doses for both treatments on Days 3 and 10. Outside of this window, water will be allowed *ad libitum*.

6.4. Randomization

A computer-generated randomization schedule will be prepared before the start of the study. At admission on Day -1, subjects will be randomly assigned in a 1:1 ratio to 1 of 2 treatment sequences: AB or BA, where Treatment A is GTX-104 (test formulation) and Treatment B is nimodipine oral capsules, RS (reference formulation).

6.5. Blinding

This will be an open-label study conducted without blinding.

6.6. Restrictions and Prohibitions

6.6.1. Birth Control

Male subjects who are nonsterilized and sexually active with a female partner of childbearing potential must agree to use a protocol-approved method of contraception from signing of the ICF, throughout the duration of the study and for 30 days after the last dose of study drug. Acceptable methods of contraception include:

- Abstinence
- Surgical sterilization of the subject or partner
- FDA-approved female hormonal contraceptives
- Intrauterine device (IUD)
- Male or female condoms with spermicide
- Diaphragm with spermicide
- Infertile partner

Female subjects of childbearing potential who are sexually active with a nonsterilized male partner must agree to use a protocol-approved method of contraception from signing the ICF, throughout the duration of the study and for 30 days after the last dose of study drug. Acceptable methods of contraception include:

- Abstinence
- Non-hormonal IUD
- Male or female condoms with spermicide
- Diaphragm with spermicide

Female subjects of nonchildbearing potential must be surgically sterile (i.e., hysterectomy, bilateral oophorectomy or bilateral tubal ligation) and at least 6 months post-surgical, or must be postmenopausal, defined as no menstrual period for at least 12 months, and confirmed by FSH level of ≥40 IU/L.

Subjects must not use oral contraceptives, hormone replacement therapy, or any estrogen-containing medications from 30 days prior to Day 1, through completion of the EOS assessments. If discontinuation of oral contraceptives by at least 30 days is not logistically possible to meet the planned study start date, the subject may be rescreened to participate in a later cohort assuming the oral contraceptive restriction is then met.

Potential subjects currently using oral contraceptives may be included in the study if they are willing to switch their birth control method to condoms and spermicide for a sufficient washout period before study entry and throughout the duration of the study, with advisement to use dual protection for 1 month after the study.

6.6.2. Prior and Concomitant Therapy

Any medications taken within 30 days of the Screening visit should be recorded as a prior/concomitant medication in the eCRF.

For any new mediations started by the subject during the study, Investigator discretion should be used regarding the potential impact of the co-administered medication on the PK parameter values.

The intention is to limit subject use of any concomitant medications during the study. Any medications used during Screening and during the study are to be recorded in the applicable eCRF page. The term “concomitant medication” encompasses prescription medications, over-the-counter (OTC) products, and herbal products used in therapeutic amounts. The name of the medication (generic name), total daily dose and route, dates of start and stop, and reason for use will be recorded in the eCRF.

Treatments that are prohibited before or during the study are shown in Table 6-1.

Table 6-1. Prohibited Prior and Concomitant Treatments

Treatment	Restricted Time Period
Alcohol	From 3 days before clinic admission through completion of EOS assessments
Nicotine or nicotine products	From 6 months before first dosing through completion of EOS assessments
Testosterone	
• administered via implant or injection	From at least 6 months before Screening through completion of EOS assessments
• administered by any other route	From at least 7 days before Screening through completion of EOS assessments
Opioids, neuroleptics, lithium, antidepressants, mood stabilizers, benzodiazepines, cognitive enhancers, centrally acting antihypertensives, GABA-agonists	From at least 3 months before Screening through completion of EOS assessments
Drugs that could interfere with glucuronidation, or that are known to exacerbate renal dysfunction (e.g., amphotericin B, aminoglycoside antibiotics, trimethoprim, NSAIDs, antihypertensive agents that excessively reduce cardiac output or systemic blood pressure); and OTC medications, including acetaminophen, topical medications, vitamins, and nutritional supplements (note: may be allowed at the discretion of the Investigator).	From at least 2 weeks before Day 1 through completion of EOS assessments
Oral contraceptives, hormone replacement therapy, or any estrogen-containing medications	From at least 30 days before Day 1 through completion of EOS assessments

Treatment	Restricted Time Period
MAOIs, prescription medicines, herbal preparations (e.g., St John's wort), or receive any immunizations	From at least 30 days before Day 1 through completion of EOS assessments
Any strong or moderate inhibitor of CYP3A4	Within 14 days or 5 half-lives, whichever is longer, of dosing on Day 1
More than 1 dose of a CYP3A4 inducer	Within 30 days preceding clinic admission
Experimental drug and/or experimental medical device	From 28 days before Day 1 or <5 times the drug's elimination half-life, whichever is longer, through completion of EOS assessments

CYP = cytochrome P450; EOS = End of Study; GABA = gamma-aminobutyric acid; MAOI = monoamine oxidase inhibitor; NSAID = nonsteroidal anti-inflammatory drug; OTC = over-the-counter

Nausea and/or vomiting deemed due to the study drug will be managed per established medical practices, which may include treatment with an antiemetic (promethazine) at the Investigator's discretion (see Section 8.15.1).

6.6.3. Subject Activity

Subjects will not be permitted to participate in any strenuous exercise, sunbathing, use of tanning booth, or significant sun exposure within 3 days prior to clinic admission through completion of the EOS assessments.

6.6.4. Treatment Compliance

Subjects will receive all study drugs in a supervised clinical study unit, and study drugs will be administered by study personnel throughout the study (as specified in Section 6.2). The date and time of study drug administration will be recorded in the eCRFs. Compliance will be monitored by study personnel at the site and will be recorded on source documents, the accountability record, and in the eCRFs.

Any deviations in treatment compliance will be recorded along with an explanation for the deviation. An overall accountability of study drug will be performed by the Study Monitor.

7. STUDY DRUG MATERIALS AND MANAGEMENT

7.1. Packaging and Labeling

7.1.1. Investigational Product

Study drug (GTX-104) will be packaged in 5-mL amber glass vials containing 10 mg of nimodipine USP (2 mg/mL). Each vial of GTX-104 provided by the Sponsor will be packaged in a single carton with a single-panel, computer-generated label (printed and approved by the Sponsor) that will include at minimum the following information:

- Sponsor identification details
- Protocol number
- Required cautionary statements
- Drug identification, including lot number
- Manufacturer name and address
- Date of expiry or retest

- Storage conditions

All text will be in English and French.

7.1.2. Reference Product

Oral nimodipine will be supplied by the study center in commercial packages of nimodipine capsules, RS (BIONPharma, Inc.). Each oblong, white, opaque, liquid filled, soft-gel capsule will be printed with NM1 in black ink, and will contain 30 mg of nimodipine and the following inactive ingredients: gelatin, glycerin, hypromellose, iron oxide black, kosher glycerin, mannitol, peppermint oil, polyethylene glycol, propylene glycol, sorbitol, sorbitol anhydrides, and titanium dioxide.

The package label principal display panel will include the following information:

NDC 69452-0209-20 (100 capsule count), 69452-0209-13 (30 Capsule Count)
BIONPharma Inc.
Nimodipine Capsules, 30 mg
Rx only

7.2. Storage and Accountability

7.2.1. Investigational Product Storage

Study drug (GTX-104) will be shipped in a validated shipping container at ambient temperature. At the study center, the study drug will be stored in the manufacturer's original package at 25°C (77°F) with excursions permitted to 15°C to 30°C (59°F to 86°F). The study drug is stable at 25°C (77°F) for up to 9 months at 60% relative humidity.

7.2.2. Reference Product Storage

Nimodipine oral capsules (RS) will be stored in the manufacturer's original package at 20°C to 25°C (68°F to 77°F) [see USP Controlled Room Temperature], with excursions permitted to 15ºC to 30°C (59ºF to 86°F). Capsules will be protected from light and freezing.

7.2.3. Accountability

Investigational products are for investigational use only, and the study drug supplied for this study is intended for use only within the context of this study. The study drug supplied for this study should be stored in a secure place and maintained under adequate security until dispensed for subject use or returned to the Sponsor.

The Investigator, Pharmacist, or their designee, will verify that study drug supplies are received intact and in the correct amounts by signing and dating the investigational product receipt log. The person receiving the supplies must verify that the shipment contains all the items noted in the shipment inventory. Any damaged or unusable study drug in a given shipment will be documented in the study files. The Investigator must notify the Sponsor or designee of any damaged or unusable investigational product supplied to the Investigator's site.

The site will maintain a Drug Inventory Log (including, but not limited to, the following: lot number, number of vials received, and number of vials dispensed). The site will also maintain subject-specific drug dispensing logs for the study drug and the reference product.

An overall accountability of investigational product will be performed and verified throughout the study and at the site closeout visit. Upon completion of the study, copies of the investigational product accountability records will be returned to the Sponsor. By signing the Investigator Agreement page of this protocol, the Investigator or named sub-investigator agrees not to supply study drug to any person(s) not enrolled in the study.

7.3. Study Drug Dispensing

Study drug will be dispensed by a trained pharmacy technician under the supervision of a Pharmacist or suitably-qualified designee per the schedule outlined in Section 6.2. Study drug will be supplied only to subjects participating in the study. The Investigator agrees to neither administer the study drug from, nor store it at any location other than the study site agreed upon with the Sponsor.

7.4. Bioavailability Sample Retention

In compliance with 21 CFR 320.38 and the U.S. FDA Guidance for Industry on retention of BA samples, reserve investigational product samples will be retained for at least 5 years following the date of marketing application approval or at least 5 years following the date of completion of the study in which the sample was used.

8. STUDY EVALUATIONS

All procedures described in this section will be performed by a qualified member of the clinical research staff.

8.1. Informed Consent

The Investigator will obtain informed consent from each subject enrolled in the study, in accordance with the Declaration of Helsinki, the current version of the International Conference on Harmonisation (ICH) guidelines and the laws and regulations of the country in which the investigation is being conducted. An appropriately constituted IRB must approve the ICF to be used by the Investigator.

The Investigator or qualified designee will discuss the study with the subject, address any questions and/or concerns that the subject may have, and if there is continued interest, will secure written informed consent for participation in the study, as well as any locally required authorization (e.g., Health Insurance Portability and Accountability Act [HIPAA]).

It is the responsibility of the Investigator to ensure that the subject has signed the ICF before any study-related procedures are performed. This includes, but is not limited to, the performance of diagnostic or therapeutic procedures and the administration of study medication.

The ICF must be signed and dated by the subject and the qualified research professional obtaining the subject’s consent. If consent is provided on the same day that any study procedure is also to be performed, the time at which consent was provided will also be recorded on the consent form or in the source documents. A subject is considered enrolled in the study when he/she has provided written informed consent.

8.2. Subject Identification Number

A SID number will be assigned at Screening and Screening evaluations may begin. The SID numbers will be assigned in sequential order and will be used to identify the subject during the Screening process and throughout study participation. Upon admission, the subjects will also be assigned a randomization number. Study drug will be dispensed according to Randomization scheme.

8.3. Demographics and Medical History

The comprehensive medical history will include: demographic information (including date of birth, gender, and self-reported race and ethnicity; both medical and psychiatric conditions; surgical history and any scheduled medical procedures; tobacco, alcohol, caffeine use; illicit drug use; blood donation history for the past 3 months; and participation in other drug or device trials in the past 28 days). The date of last menses for female subjects should be recorded. Any findings on the baseline safety assessments (at Screening) such as clinical laboratory test results, ECG findings, and physical examination will be considered medical history and would be exclusionary if deemed clinically significant based on the Exclusion Criteria, or may be documented as AEs, at the discretion of the Investigator.

The medication history will include use of any prescription and non-prescription drug, nutritional supplement or herbal product, or substance, whether taken internally or applied topically, within 30 days before Screening. Additionally, use of one or more medications described in the Exclusion Criteria within up to 6 months before Screening (Table 6-1) should also be recorded in the medication history.

8.4. Physical Examinations

Physical examinations will be completed as described in the Time and Events Schedule (Table 4-1). A complete physical examination will be performed at Screening, including assessments of the skin, head, eyes, ears, nose, throat, neck, thyroid, lungs, heart, abdomen, lymph nodes, extremities, height, and weight, and a limited neurological examination. The physical examination will not include a pelvic, breast, or rectal exam.

An abbreviated physical examination including assessment of skin, peripheral pulses, lungs and heart will be performed on admission to the CRU, and at the end of the subject’s study participation (EOS or ET). Additional assessments may be performed at the discretion of the Investigator. Any significant physical examination finding at Screening should be recorded in the subject’s medical history. If any clinically significant change is noted from Screening, it will be reported as an AE and will be followed up to resolution or upon reaching a stable end point.

8.4.1. Height, Weight, and Body Mass Index

Height will be measured only at Screening using a stadiometer or other standard device, with the subject wearing no shoes.

Body weight will be measured at Screening, on admission to the CRU, and again at the EOS participation (EOS or ET). All weights will be measured with a standard physician’s scale and with the subject wearing only light indoor clothing and no shoes.

The Screening value will be used to calculate BMI to assess eligibility, which will be calculated using the following formula: BMI (kg/m^2) = Weight (in kilograms)/Height (in $meters^2$).

8.5. Vital Signs

Vital sign measurements (SBP, DBP, pulse rate, respiration rate, and body temperature) will be obtained after the subject has been in the sitting or semi-supine position for at least 5 minutes at Screening and at the time points indicated in the Time and Events Schedule (Table 4-1).

Measurement of BP should be done on the same arm (contralateral to the arm used for IV drug administration), as is operationally feasible, throughout the study using a completely automated device consisting of an inflatable cuff and an oscillatory detection system. Manual BP readings may be obtained by a qualified clinical staff member in the event of instrument malfunction. BP measurements by oscillometric sphygmomanometer will be recorded for both Treatment A and Treatment B, prior to the first dose and 60 minutes post dose for the 8:00 AM, 12:00 PM, 4:00 PM, and 8:00 PM doses on Days 3 and 10, as described in Section 8.10.

Body temperature will be measured with an thermometer.

If the measurement of vital signs is scheduled or occur at the same time as ECG measurements or the collection of blood samples, vital signs will be measured after ECG assessments, and before the collection of any blood samples (clinical laboratory or PK) because drawing blood may affect the vital signs.

Out-of-range BP measurements at Screening may be repeated once at the discretion of the Investigator, so that up to 2 consecutive assessments are made within 1 hour. Out-of-range BP measurements on admission to the CRU, or before dosing on any other day, may be repeated up to 3 times at intervals of approximately 15 minutes if, in the Investigator's opinion, repeat measurements are appropriate.

8.6. Orthostatic Hypotension

Orthostatic hypotension is defined as a reduction of SBP of at least 20 mmHg or a drop in DBP of 10 mmHg within 3 minutes of standing (Shibao et al., 2013).

Testing for orthostatic hypotension will be conducted at Screening and at CRU admission (Day -1) using an automated system to measure SBP, DBP, and heart rate. Measurements will be taken after the subject has been supine for a minimum of 5 minutes, followed by re-measurements at 1 minute and 3 minutes with the subject in the standing position. Findings will be documented in the eCRF.

8.7. Electrocardiograms

A 12-lead ECG will be obtained from each subject at Screening and at other time points per the Time and Events Schedule (Table 4-1), after the subject has been in supine or semi-reclined position for at least 5 minutes, and before the measurement of vital signs or the collection of blood samples. The ECG measurements will be performed in triplicate and will include QTcF, RR, PR, and QRS intervals; the averages of the triplicate readings will be recorded in the eCRF.

All ECGs must be reviewed for safety by a physician or other staff member who is qualified to read and interpret the results. Each ECG tracing must be noted as “not clinically significant” (NCS) or “clinically significant” (CS), signed and dated by the reader, and appropriately recorded in both the source documents and the eCRF. An ECG recorded after Screening/admission to the CRU, which is considered CS should be recorded as an AE.

8.8. CYP3A4 Genotyping

A buccal swab or blood test will be performed for CYP3A4 genotyping at Screening.

8.9. Clinical Laboratory Evaluations

All laboratory tests will be performed on-site, by the laboratory affiliated with the Phase 1 unit. Blood and urine for clinical laboratory testing prior to enrollment will be collected after an overnight fast (at least 8 hours) and will include evaluations summarized in Table 8-1.

Venous blood will be drawn for the tests listed in Table 4-1. Because of the large number of samples to be collected, repeated venipuncture should be minimized, as well as long-term indwelling catheters. Ideally, venipuncture will be performed for Day 1 PK samples. On Day 2, only any required infusion line should be in place. On Day 3, an indwelling large gauge catheter should be placed for sample collection in the contralateral arm from any infusion line.

Subjects will be in a seated or semi-reclined position during blood collection.

Any abnormal hematology, serum chemistry, or urinalysis test result deemed CS by the Investigator will be reported as an AE and repeated, including test results obtained at EOS/ET. Repeat analyses will be performed until resolution, or until the Investigator determines that resolution of the laboratory abnormality is not expected.

If blood samples for clinical laboratory tests are scheduled to be collected at the same time as an ECG or vital signs, the ECG and/or vital signs must be obtained before the blood sample collection as drawing blood may affect the vital signs.

The total volume of blood collected for clinical laboratory tests from each subject is not expected to exceed 60 mL over the study (not including repeat laboratory tests, should these be necessary). See Appendix 1 for the estimated total volume of blood collected from each subject over the course of the study.

Table 8-1. Clinical Laboratory Evaluations

**Chemistry**		**Urinalysis**
Albumin	Glucose	Appearance and color
Alkaline phosphatase	Globulin	Blood
Alanine aminotransferase (ALT)	Iron	Glucose
Aspartate aminotransferase (AST)	Lactate dehydrogenase (LDH)	Ketone
Bicarbonate	Lipase	Protein
Bilirubin, total	Magnesium	pH
Bilirubin, direct	Phosphate	Specific gravity
Calcium	Potassium	Nitrite
Carbon dioxide	Protein, total	Bilirubin
Chloride	Sodium	Urobilinogen
Cholesterol, total	Triglycerides	Microscopy
Creatine phosphokinase (CPK)	Urea (Blood urea nitrogen; BUN)	White blood cells (WBC)
Creatinine	Uric Acid	Red blood cells (RBC)
Gamma-glutamyl transpeptidase (GGT)		Hyaline casts
**Hematology**		Waxy casts
Hematocrit	WBC count	WBC casts
Hemoglobin	Differential	RBC casts
Mean corpuscular hemoglobin (MCH)	Basophils	Epithelial cells
MCH concentration (MCHC)	Eosinophils	Crystals
MC volume (MCV)	Lymphocytes	Mucous threads
RBC count	Monocytes	Bacteria
	Neutrophils	Yeast
	Platelets	
**Serology (screening only)**		**Urine Alcohol/Drug Screen**
Human immunodeficiency virus (HIV)<br>Hepatitis B surface antigen<br>Hepatitis C virus (HCV)		Amphetamines, barbiturates, benzodiazepines, cocaine, cotinine, ethanol, marijuana (THC), methadone, opioids, phencyclidine, tricyclic antidepressants
**Other:**		**Coagulation:**
HbA1c (glycosylated hemoglobin): (diabetes screen)<br>Serum β-hCG pregnancy test (for female subjects of childbearing potential)<br>FSH (for postmenopausal female subjects)<br>CYP3A4 genotype determination<br>COVID-19 virus		Prothrombin time (PT) with INR<br>Activated partial thromboplastin time (PTT)

CYP = cytochrome P450; FSH = follicle-stimulating hormone; Hb = hemoglobin; hCG = human chorionic gonadotropin; INR = international normalized ratio; THC = tetrahydrocannabinol

8.9.1. Reference Ranges

Up-to-date reference ranges for the above investigations must be obtained for the laboratory performing analyses prior to the start of the study and must be updated as appropriate during the course of the study.

8.9.2. Sample Collection and Storage

Clinical laboratory samples of the appropriate biological matrix will be collected from each subject and processed by qualified clinic staff for analysis on-site, at the laboratory affiliated with the CRU.

If blood samples for clinical laboratory tests are scheduled to be collected at the same time as an ECG or vital signs, then the ECG and vital signs must be obtained before the blood sample collection because drawing blood may affect the ECG tracing or vital signs.

Following collection, the handling, centrifugation, cooling, and storage of the laboratory samples will be guided by the standard operating procedures of the CRU.

8.9.3. Laboratory Results Review

The Investigator or medically-qualified designee will review the results and comment on all abnormal values on the laboratory results sheet, identifying those that are NCS as well as those that are CS. The Investigator or medically-qualified designee will sign and date the laboratory results sheet to indicate that the review has taken place.

8.9.4. Good Laboratory Practice Compliance

Confirmation of compliance with Good Laboratory Practice (GLP) will be required from the bioanalytical laboratory and the safety laboratory prior to the start of the study. Documented evidence of GLP compliance may be a statement of compliance issued by the appropriate national authority or details of accreditation by a recognized organization.

8.10. Blood Pressure Monitoring

Monitoring and assessment of BP will be conducted by the study site per standard operating procedures.

For both Treatment A and Treatment B, BP will should be recorded via oscillometric sphygmomanometer at baseline (prior to first dose for both treatment arms) and 60 minutes post dose for the 8:00 AM, 12:00 PM, 4:00 PM, 8:00 PM doses on Days 3 and 10. The same arm is to be used for BP determinations will be used for all BP determinationswith methods consistent with recommendations from the American College of Cardiology and American Heart Association (Whelton et al., 2018).

Baseline (prior to first dose, both treatment arms) assessment will occur within 1 hour prior to initiation of infusion or oral dose. Subjects will be placed in the same position to be used for drug administration, and the same arm to be used for BP determinations following dosing will be used for baseline BP determinations. The proper cuff size will be used, and the same cuff size will be used for all BP determinations associated with the current drug treatment. The arm is to be extended and supported at the same level as the heart. Three sequential BP determinations will be made within a 15-minute time span within 1 hour prior to drug administration. The baseline blood pressure will be the mean of the three BP determinations.

This procedure will be repeated with single measurements 60 minutes post dose on Days 3 and 10 for the 8:00 AM, 12:00 PM, 4:00 PM, and 8:00 PM doses. Subjects will be asked to refrain from strenuous activity for the duration of this assessment.

8.11. Pharmacokinetic Assessments

8.11.1. Pharmacokinetic Blood Sample Collection

Blood samples for PK analysis will be taken based on Treatment. For subjects who terminate early from the study, a blood sample for PK analysis should be collected; the PK time point closest to the ET time and the relative dosing day shall be recorded in the appropriate eCRF.

A total of 116 blood samples (approximately 3 mL each) will be collected from each subject for PK determination. The estimated total blood volume per subject for PK analysis is expected to be 394 mL. See Appendix 1 for the estimated total volume of blood collected from each subject over the course of the study.

8.11.1.1. Treatment A (Nimodipine Infusion) Pharmacokinetic Blood Sample Collection

Note: PK and clinical venous blood sampling should occur in the arm opposite to infusion, and the infusion line should not be used for PK sampling.

- Day 1: Pre-dose (time 0, before initiation of the first [8 AM] infusion), and at 0.33, 0.5, 0.75, 1, 1.33, 2, 3, and 4 hours post initial (8 AM) dose.
- Starting on Day 3, blood samples for PK should be taken at: 48 (prior to initiation of the 8 AM 4 mg dose), 48.33, 48.5, 48.75, 49, 49.33, 50, 51, 52, 52.33, 52.5, 52.75, 53, 53.33, 54, 55, 56, 56.33, 56.5, 56.75, 57, 57.33, 58, 59, 60, 60.33, 60.5, 60.75, 61, 61.33, 62, 63, 64, 64.33, 64.5, 64.75, 65, 65.33, 66, 67, 68, 68.33, 68.5, 68.75, 69, 69.33, 70, 71, 72-hours post-start of first infusion.
- Note, for timepoints that are scheduled at the same time as the 4 mg dose (48, 52, 56, 60, 64, 68, and 72 hours post-start of infusion), blood for PK analysis should be taken before initiation of the 30-minute infusion. For the 0.5, 48.5, 52.5, 56.5, 60.5, 64.5, and 68.5-hour timepoints, blood samples for PK analysis should be collected after completion of the 4 mg infusion.

8.11.1.2. Treatment B (Oral Nimodipine) Pharmacokinetic Blood Sample Collection

Note: Subjects will take a total of 18 oral doses during the 72 hours of treatment, administered q4h starting at approximately 8 AM on Day 1 (e.g., 8 AM, 12 PM, 4 PM, 8 PM, 12 AM, 4 AM).

- Day 1: Pre-dose (time 0, before the first [8:00 AM] dose), and at 0.33, 0.5, 0.75, 1, 1.33, 2, 3, and 4 hours post initial (8:00 AM) dose.
- Day 3, blood samples for PK should be taken at: 48 (prior to initiation of the 8:00 AM 60 mg dose), 48.33, 48.5, 48.75, 49, 49.33, 50, 51, 52, 52.33, 52.5, 52.75, 53, 53.33, 54, 55, 56, 56.33, 56.5, 56.75, 57, 57.33, 58, 59, 60, 60.33, 60.5, 60.75, 61, 61.33, 62, 63, 64, 64.33, 64.5, 64.75, 65, 65.33, 66, 67, 68, 68.33, 68.5, 68.75, 69, 69.33, 70, 71, 72-hours since the first dose.
- Note, for timepoints that are scheduled at the same time as the 60 mg oral dose (48, 52, 56, 60, 64, 68, and 72 hours since first dose), blood for PK analysis should be taken before oral dose intake.

8.11.2. Pharmacokinetic Assessment Windows

Deviations from the scheduled sample collection time of ±5 minutes for samples at 0.25 through 3 hours post-dose and ±10 minutes for all samples at >3 hours post-dose will not be considered protocol deviations.

Pre-dose (time 0) blood samples must be collected before dosing. The exact time of study drug administration (oral dose time, and both start and stop times for both the 30-minute IV infusions and the continuous IV infusion) and the blood sampling for PK assessments will be recorded in the eCRF.

8.11.3. Bioanalytical Analysis

From each blood sample, plasma will be harvested and stored frozen (at approximately –70°C, or approximately –20°C if –70°C is not available), at the bioanalytical laboratory. Details of PK blood sample collection, processing, storage, and shipping will be provided in a separate laboratory manual.

Analysis of plasma concentrations of nimodipine will be performed using a validated method of liquid chromatography with tandem mass spectrometry (LC-MS/MS).

8.12. Safety

The safety assessments will include the nature, frequency, and severity of AEs by treatment group, laboratory safety parameters (hematology, biochemistry, coagulation, and urinalysis), SBP, DBP, other vital signs (pulse rate, respiration, and temperature), physical exam findings, and 12-lead ECGs.

Details of AE assessments are provided in Section 9.

8.13. Concomitant Medication

At Screening and at the time points indicated in the Time and Events Scheduled (Table 4-1), a qualified member of the research staff will query subjects about medication (prescription or non-prescription), herbal remedies, nutritional supplements, or topically applied treatments the subject is using or has used. Concomitant medications will be coded using the World Health Organization drug dictionary (WHO-DDE, Version March 2017, Anatomical Therapeutic Chemical classes [ATC Level 4]). This information will be documented in the source documents and eCRFs and used to determine whether the subject qualifies to continue in the study.

8.14. Assessment Windows

All assessments designated as pre-dose should be completed prior to the dose time as recorded in the eCRF.

PK assessment windows are provided in Section 8.11.2.

Vital sign measurements: ±15 minutes from scheduled time point. At any point that requires measurement of vital signs at the same time as blood collection, vital signs (especially BP) and/or the ECG should be collected BEFORE blood is drawn.

8.15. Other Study Procedures

8.15.1. Nausea or Vomiting

If nausea or vomiting occur, treatment with promethazine is permitted. If resolved, the subject can remain in the study.

8.15.2. Potential Pregnancies

If it is determined that a subject is pregnant based on the laboratory results at Screening or at admission to the CRU, the site should screen fail the subject and refer her to her physician (providing her a copy of any relevant laboratory reports). If a subject is found to be pregnant any time after admission to the CRU until 30 days after the last dose of study medication, it should be reported to the Investigator and followed through the completion of pregnancy and 6 to 12-month follow-up of the newborn, or as appropriate.

8.15.3. Unplanned infusion interruptions:

See Table 8-2 for summary of algorithm. If interruptions occur to drug administration due to IV-specific issues (i.e., pump failure, infiltration, unintentional IV removal) the infusion will be restarted as quickly as practical. If the interruption(s) occurs during the initial 30 minute, high-rate infusion period, and the interruption(s) total to <3 minutes, then the high-rate infusion time and 0.5 hour blood sample collection time will extend accordingly (e.g., if the high-rate IV infusion is interrupted at 15 minutes, and is restarted in three minutes, the infusion is stopped from 15 minutes to 18 minutes, so the high-rate infusion period will continue for an additional 3 minutes, to 33 minutes), at which point (33 minutes) the nominal 0.5 hour sample will be collected. The timing of these events will be noted in the eCRF. If the experienced interruptions to the high-rate infusion on Day 1 is greater than or equal (≥) to 3 minutes for any given q4h dosing cycle on Day 1, the subject will be released from the CRU for that day. The subject may return to the CRU the following day, at which point the Day 1 will be repeated. This deviation will be recorded by the principal investigator.

If infusion interruption occurs during the high-rate infusion on Days 2, the infusion will be restarted as quickly as possible. Infusion interruptions during high-rate infusion on Day 2 will not result in withdrawal of the subject.

On Day 3, similar criteria as for Day 1, will apply if the interruption(s) total to <3 minutes during the high-rate infusion period for any given q4h dosing cycle, i.e, the timing of the end of infusion sample will be adjusted so that it occurs at the end of infusion at this rate (e.g., if the infusion is interrupted for 3 minutes, the end of infusion sample will be collected at 33 minutes after the start of the high-rate infusion, rather than 30 minutes). The nominal time for other samples (e.g., 48.75, 49, 50 hours) will be unchanged. If the total interruption during during any given q4h dosing cycle exceeds 3 minutes on Day 3, the patient will be withdrawn from the study and replaced. Infusion interruptions during high-rate infusion on Day 2 will not result in withdrawal of the subject.

On Day 3 if interruptions occur to the drug administration due to IV-specific issues during the low-rate infusion period the infusion will again be restarted as quickly as possible. If the total duration of interruptions of the low-rate infusion during Day 3 exceeds 30 minutes, during any given q4h dosing cycle that patient will be withdrawn from the study and will be replaced. Interruptions of infusion during low-rate infusion on Days 1 and 2 will not result in withdrawal

of the subject. Interruptions of infusion during the low-rate infusion will not alter sample collection times. See Table 8-2 for summary of algorithm.

Table 8-2. Action to be Taken for Infusion Interruptions

	**Day 1**	**Day 2**	**Day 3**
Interruption in high-rate infusion	Restart infusion as quickly as practical If total interruption ≥3 minutes in any q4h dosing cycle, discharge subject, restart the following day. If total interruption <3 minutes total interruption, adjust nominal 0.5-hour sample to occur at the end of infusion. Other nominal time points will be unchanged. Document timing of infusion interruption in eCRF.	Restart infusion as quickly as practical. Document timing of infusion interruption in eCRF.	Restart infusion as quickly as practical<br>If total interruption ≥3 minutes in any q4h dosing cycle, subject to be withdrawn and replaced. If total interruption <3 minutes for any given q4h dosing cycle, adjust nominal 0.5-hour sample to occur at the end of infusion. Other nominal time points will be unchanged. Document timing of infusion interruption in eCRF.
Interruption in low-rate infusion	Restart infusion as quickly as practical. Document timing of infusion interruption in eCRF.	Restart infusion as quickly as practical. Document timing of infusion interruption in eCRF.	Restart infusion as quickly as practical If total interruption >30 minutes in any q4h dosing cycle, subject to be withdrawn and replaced. Document timing of infusion interruption in eCRF.

9. ASSESSMENT OF SAFETY

Any AE reported from the time written informed consent is obtained until completion of the final EOS or ET assessments, regardless of the association with study drug, shall be recorded in the source documents and on the appropriate eCRF page. The AE time of onset (24-hour clock), duration, causality, action taken, and follow-up procedures will be recorded.

9.1. Definitions

9.1.1. Adverse Event

An AE is any untoward medical occurrence associated with the use of a drug in humans, whether or not considered drug related (Code of Federal Regulations [CFR] Title 21, Part 312.32[a]). The occurrence does not necessarily have to have a causal relationship with this treatment. An AE can therefore be any unfavorable and unintended sign (for example, an abnormal laboratory

finding), symptom, or disease (new or exacerbated) temporally associated with the use of a drug, whether or not considered related to the study drug.

TEAEs are events that are not present at baseline, or if present at baseline, have worsened in severity after the administration of the first dose of any drug in the study regardless of causality.

9.1.2. Life-threatening Adverse Event

An AE is considered "life-threatening" if, in the view of either the Investigator or Sponsor, its occurrence places the subject at immediate risk of death. It does not include an AE that, had it occurred in a more severe form, might have caused death.

9.1.3. Serious Adverse Event

An AE is considered "serious" (that is, an SAE) if, in the view of either the Investigator or Sponsor, it results in any of the following outcomes:

- Death.
- A life-threatening AE (at risk of death at the time of the event).
- Inpatient hospitalization or prolongation of existing hospitalization.
- A persistent or significant disability/incapacity or substantial disruption of the ability to conduct normal life functions.
- A congenital anomaly/birth defect.

Important medical events that may not result in death, be life-threatening, or require hospitalization may be considered serious when, based upon appropriate medical judgment, they may jeopardize the subject and may require medical or surgical intervention to prevent one of the outcomes listed in the SAE definition.

9.2. Recording

Any AE/SAE reported from the time written informed consent is obtained, and after Screening until the completion of the final EOS or ET assessments, regardless of the association with the study drug, shall be recorded in the source documents and on the appropriate eCRF page.

The Investigator should review all documentation (e.g., hospital progress notes, laboratory, or diagnostic reports) relative to the event being reported. The Investigator will then record all relevant information regarding an AE/SAE on the appropriate eCRF page. It is not acceptable for the Investigator to send photocopies of the subject's medical records in lieu of completion of the appropriate AE/SAE on all appropriate study documents. However, there may be instances when the Sponsor (or its designee) requests copies of medical records for certain cases. In this instance, all subject identifiers will be blinded on the copies of the medical records prior to submission to the Sponsor (or its designee).

For each AE, the Investigator will evaluate and report the onset (date and time), resolution (date and time), intensity, causality, action taken, serious outcome (if applicable), and whether or not it caused the patient to discontinue the study. The Investigator will attempt to establish a diagnosis of the event based on signs, symptoms, and/or other clinical information. In such cases, the diagnosis should be documented as the AE/SAE and not the individual signs and symptoms. In order to classify AEs and diseases, preferred terms will be assigned by the Sponsor or its designee to the original terms entered on the eCRF, using Medical Dictionary for Regulatory Activities (MedDRA) terms.

9.3. Severity

The Investigator will assess the intensity of each AE/SAE based on his/her clinical judgment. The intensity of each event should be assigned to one of the following categories:

Mild: An event that is easily tolerated by the subject, causing minimal discomfort and not interfering with everyday activities.

Moderate: An event that is sufficiently discomforting to interfere with normal everyday activities.

Severe: An event that prevents normal everyday activities. Subject may experience intolerable discomfort or pain.

An AE that is assessed as severe should not be confused with an SAE. Severity is a category utilized for rating the intensity of an event; both AEs and SAEs can be assessed as severe. An event is described as 'serious' when it meets one of the pre-defined outcomes as described in Section 9.3.

9.4. Clinical Laboratory Adverse Events

The Investigator will exercise medical judgment in deciding whether abnormal laboratory values, ECGs, or other assessment are CS.

Abnormal laboratory findings (e.g., clinical chemistry, hematology, coagulation, urinalysis) or other abnormal assessments (e.g., ECGs, vital signs) that are judged by the Investigator as CS will be recorded as AEs or SAEs if they meet the definition of an AE or SAE (as defined in Section 9.1).

9.5. Timing

The reporting period for all AEs begins with the subject signing the ICF and ends with the EOS or ET assessments.

9.6. Follow-up

After the initial AE/SAE report, the Investigator is required to proactively follow each subject and provide further information to the Sponsor (or its designee) on the subject's condition. All AEs and SAEs documented as ongoing will be followed and reviewed until they resolve, they become medically stable in the Investigator's opinion, or 4 weeks have passed after the last visit, whichever occurs first.

9.7. Relationship or Causality

The Investigator will use clinical judgment to assess AEs for relationship to the study drug, and will also consult the Investigator's Brochure for the study drug and/or the product or prescribing information for the concomitant medications that are marketed products in making his/her assessment.

The assessments will be recorded on the source documents and eCRF, using the categories defined below:

Unlikely: A clinical event, including laboratory test abnormality, with a temporal relationship to drug administration which makes a causal relationship improbable, and in which other drugs, chemicals, or underlying disease provide plausible

explanations. For the purpose of this protocol, the term “unlikely” will be considered an AE not related to study drug.

Possible: A clinical event, including laboratory test abnormality, with a reasonable time sequence to administration of the drug, but which could also be explained by concurrent disease or other drugs or chemicals. Information on drug withdrawal may be lacking or unclear.

Probable: A clinical event, including laboratory test abnormality, with a reasonable time sequence to administration of the drug, unlikely to be attributed to concurrent disease or other drugs or chemicals, and which follows a clinically reasonable response on withdrawal.

If the relationship between the AE/SAE and the study drug is determined to be “possible” or “probable” the event will be considered to be related to the study drug for the purposes of expedited regulatory reporting.

9.8. Reporting Adverse Events

All SAEs, whether or not unexpected or considered to be associated with the use of the drug, and regardless of the timing of their occurrence after the first dose of study drug, will be reported to the Sponsor’s Medical Monitor or designee by the Investigator through telephone, fax, or email within 24 hours of discovery, using the form provided by the Sponsor. The Medical Monitor or designee will then notify the Sponsor and advise the Investigator regarding the nature of any further information or documentation that is required. Contact information for SAE reporting is provided in Table 9-1.

Table 9-1. Contact Information for SAE Reporting

	Medical Monitor	Sponsor Contact
	Judith Johnson	S. George Kottayil
Phone	(978) 618-7770	(646) 668-4502
Fax	+1 919-591-0004	848-209-9337
Email	jjohnson@safeharborpv.com	gkottayil@gtrx.com

The Investigator (or designee) will complete an SAE form for the initial report (eCRF or paper). The initial SAE form must be as complete as possible, including details of the illness(es) and other information relevant to the SAE, and include at least the following:

- Study identifier (protocol number)
- Study center or site number
- Subject number (SID)
- Full description of the event, including other relevant information (e.g., diagnostic information that will assist in understanding the event)
- Date of event onset
- Reason the event was classified as serious
- Current status of event
- Whether or not study drug was discontinued
- Investigator’s assessment of the relationship between the event and study drug

Additionally, the Investigator (or designee) will provide the Medical Monitor with any significant new information related to the SAE that he/she receives at any time after the initial report by completing one or more follow-up SAE forms within 24 hours after the Investigator obtains the new information, and following the same procedure as for the initial SAE report.

For all SAEs involving a hospitalization, the Investigator (or designee) will obtain copies of the hospital discharge summary and any relevant diagnostic documents (e.g., emergency room reports, radiology reports, significant laboratory test results), and forward them to the Medical Monitor as soon as available.

For all fatal SAEs, the Investigator (or designee) will obtain a copy of the autopsy report, if an autopsy was performed, and forward the report to the Medical Monitor as soon as possible.

Any SAE occurring in a subject after the EOS or ET assessments that comes to the attention of the Investigator and is considered to be causally related to the study drug will also be communicated to the Sponsor, following the same instructions as for SAEs occurring during the study. The Investigator must inform the IRB immediately regarding any AE (does not have to be causally related) that is both serious and unexpected; or that represents a series of AEs that, on analysis, is unanticipated, or occurs at an unanticipated frequency, or otherwise represents an unanticipated safety risk to the study subject. The IRB may subsequently choose to modify the informed consent or request changes to the protocol.

All SAEs must be followed with appropriate medical management until resolved or until considered chronic and stable or otherwise explained.

All paper documents relating to the SAE (e.g., the original SAE form and fax transmission confirmation, copies of any supporting documentation, all SAE-related correspondence with the IRB) will be retained in the site's study file. Copies of all documents will be forwarded to the Study Monitor.

9.9. Stopping Rules

The Investigator or medically-qualified designee must contact the Sponsor immediately to discuss whether to suspend dosing if he/she believes AEs and/or laboratory abnormalities indicate that continued dosing of subsequent subjects would not be tolerated or would jeopardize the subjects' safety. The Sponsor may suspend dosing at any time for any reason.

Factors that must be considered for suspension of dosing include the frequency, severity, CS, possible causality, and anticipated reversibility of all observed AEs and/or laboratory abnormalities for each specific subject group. If any CS, treatment-related SAE is observed in at least 1 subject, dosing of subsequent subjects will be stopped pending a safety review of the data and a consideration of the appropriate course of action by Sponsor and Investigator. The IRB will be notified if dosing is suspended.

The study will be immediately suspended and no additional doses of study drug will be administered if one or more subjects develop any of the following SAEs deemed to be possibly or probably attributable to study drug by the Investigator and/or Sponsor Medical Monitor, based upon close temporal relationship or other factors:

- Death
- Serious anaphylaxis characterized by severe angioedema, hypotension, shock, bronchospasm, hypoxia, or respiratory distress

- Seizure in any subject
- QTcF ≥500 msec and confirmed on repeat ECG
- 1 subject with serum alanine aminotransferase (ALT) or aspartate aminotransferase (AST) >5x above upper limit of normal (ULN), or serum total bilirubin >2x ULN, or serum creatinine >2x ULN
- 2 subjects with >3x ULN ALT or AST, or serum total bilirubin >1.5x ULN, or serum creatinine >1.5x ULN

In addition, dose hold or discontinuation of IV GTX-104 dosing for any subject may occur if changes in BP and/or heart rate are noted, as described in Appendix 2.

Vital signs can be repeated, as appropriate, to confirm values and rule out extraneous results. Determination of early termination will be made based on clinical findings at the Investigator's discretion. If the IV infusion is stopped due to the above-referenced hemodynamic criteria, then the subject will be discontinued from the study.

The BP and heart rate criteria in Appendix 2 will be used once dosing has commenced. The vital signs outlined in the Inclusion/Exclusion Criteria (Section 5.1, Section 5.2) will be used for Screening and baseline evaluations only.

10. DATA ANALYSIS AND STATISTICAL CONSIDERATIONS

10.1. General Considerations

A comprehensive PK analysis plan (PKAP) and statistical analysis plan (SAP) that fully specifies the statistical methodology (including table, figure, and listing formats) for all aspects of the planned analyses will be developed by the Sponsor or Sponsor's designee. The PKAP and SAP will be developed as part of the pre-results clinical study report (CSR) and approved after the protocol is finalized. No database may be locked, or analyses completed until the SAP and PKAP have been approved. Additional unplanned analyses may be required after all planned analyses have been completed. Any unplanned analyses will be clearly identified in the CSR.

The SAP will provide a detailed description for the handling of missing data, patient eligibility criteria for the analysis, and statistical methodology for the data summary and analysis of safety. This protocol describes key analyses as currently contemplated. If differences occur between analyses described in the SAP and the current protocol, those found in the SAP will assume primacy.

All non-PK statistical reporting will be performed using the validated software SAS® for Windows version 9.4 or higher (SAS Institute, Inc., Cary, NC, USA), unless otherwise specified.

Individual subject data will be presented by subject in data listings. Data listings will include all data collected from the initial Screening visit up to the EOS for all subjects randomized. Both absolute values and change from baseline values for each subject will be given where applicable. Data listings will be sorted by treatment, SID, and time point. Screen failures will be included in a separate listing.

Continuous variables will be summarized using number of non-missing observations, mean, standard deviation (SD), median, minimum, and maximum; categorical variables will be summarized using the frequency count and the percentage of subjects in each category. In

addition to the descriptive summaries, pertinent data listings will be provided to facilitate case studies.

10.2. Determination of Sample Size

A model-based approach was used to estimate power and sample size. Simulated subjects (n=1000) were randomized to a two-period crossover with two sequences (AB and BA) where Treatment A was GTX-104 and Treatment B was oral nimodipine. Incorporated sample collection times are outlined in the study design (Section 4.1). Day 3 simulated data were used for the AUC power analysis. The estimate for the within subject variance (on the log scale) came from a mixed effects model that included fixed effects for sequence, period, and treatment and a random effect for subject. Under the hypothesis that the true geometric mean ratio (GMR) is 1 for GTX-104 compared to oral nimodipine, a total of 49 subjects was estimated to provide power of 0.85 (C_{max} on Day 1) and >0.99 ($AUC_{Day\ 3,\ 0\text{-}24hr}$), respectively, to assess relative BA in a two-period, two-sequence crossover study.

A minimum of 60 subjects was therefore selected for enrollment to allow at least 50 subjects completing (allowing for some subject attrition). Additional subjects may be enrolled if subject withdrawal requires additional subjects to reach a total of 50 completers.

10.3. Analysis Populations

Safety population: The Safety Population will consist of all subjects who receive at least 1 dose of study drug.

PK Population: The PK Population will consist of all subjects who receive at least 1 dose of study drug and have evaluable PK data.

Analysis Population: The Analysis Population will consist of those subjects in the PK population who are evaluable for both Treatment A (test) and Treatment B (reference).

10.4. Demographics and Baseline Characteristics

Demographic and other baseline characteristics will be summarized by treatment group.

10.5. Pharmacokinetic Analyses

A PKAP will be developed based on the latest version of the clinical protocol. No database may be locked, or analyses completed until the PKAP has been approved. The PKAP will provide a detailed description of the PK methodology, parameters, planned analyses, and tables, listings, and figures. This protocol describes key analyses as currently contemplated. If differences occur between analyses described in the PKAP and the current protocol, those found in the PKAP will assume primacy.

All PK parameters will be derived by noncompartmental analysis of the concentration-time data utilizing a validated installation of WinNonlin®, (Certara), version 6.4 or later. The models will be appropriate for plasma data from IV and oral administration. Actual sampling times will be used to calculate the PK parameters. The linear trapezoidal method will be used to integrate the concentration-time profile data.

10.5.1. Interim Pharmacokinetic Analysis

An interim analysis may be conducted after ~20 subjects complete both periods to assess the within subject variability and the observed mean exposures. Individual nimodipine PK parameters will be calculated using noncompartmental analyses and will include C_{max} on Day 1

for the first dose, $AUC_{Day\ 3,\ 0\text{-}24\ hr}$, C_{max} on Day 3, CL (for IV), and CL/F (for oral doses) at a minimum. The sample size may be increased by selecting an additional subjects for up to 100 to account for larger than anticipated variance(s) in $AUC_{Day\ 3,\ 0\text{-}24hr}$ or Cmax on Day 1 of first dose. If mean exposures are significantly dissimilar, the Treatment A dosing regimen may be modified. Any dose modifications would be described and justified in a protocol amendment.

10.5.2. Final Pharmacokinetic Analysis

10.5.2.1. Pharmacokinetic Parameters

Individual nimodipine PK parameters will be calculated using noncompartmental analyses and will include C_{max} on Day 1 of first dose, $AUC_{Day\ 3,\ 0\text{-}24hr}$, C_{max} on Day 3 for each of the 6 doses, CL (for IV), and CL/F (for oral doses) at a minimum. Individual PK parameters will be summarized by treatment using descriptive statistics. Additional PK parameters may be calculated to fully characterize the PK profile of nimodipine. The PK population will be used.

10.5.2.2. Calculation of Relative Bioavailability

To assess the relative BA of GTX-104 (Test) and nimodipine oral capsules, RS (Reference), an analysis of variance (ANOVA) model, with treatment, sequence, and period as fixed effects and subject nested within sequence as a random effect, will be performed on natural log-transformed $AUC_{Day\ 3,\ 0\text{-}24hr}$ and C_{max} (from the first dose of Day 1) from both treatment periods. The differences in the least squares means and associated 90% confidence intervals (CIs) will be back-transformed to provide the geometric ratios (Test/Reference) along with the 90% CIs for the ratios. The Analysis Population will be used. C_{max} geometric means for Day 3 will be described but no formal testing will be done to compare the oral and IV regimens.

10.6. Safety Analysis

All safety data will be listed by subject. TEAEs will be summarized for each treatment by system organ class, preferred term, severity, and relationship to test article. Observed values and changes from baseline for clinical laboratory test data, safety ECGs, physical examination results, and vital signs will be summarized using appropriate descriptive statistics. Potential future analyses will be detailed in a standalone SAP. The Safety Population will be used.

11. QUALITY CONTROL AND QUALITY ASSURANCE

11.1. Study Monitoring

Before an investigational site can enter a subject into the study, a representative of the Sponsor will visit the investigational study center to determine the adequacy of the facilities, and to discuss with the Investigator and other personnel their responsibilities with regard to protocol adherence, and the responsibilities of the Sponsor or its representatives. This will be documented in a Clinical Study Agreement between the Sponsor and the Investigator.

All aspects of the study will be carefully monitored by the Sponsor, or their designees, with respect to current Good Clinical Practice (GCP) and standard operating procedures for compliance with applicable federal regulations. In addition, the Sponsor's Quality Assurance (QA) unit may audit the study center to assure compliance with GCP (see Section 11.2).

The Investigator will be responsible for the following:

- Monitoring study conduct to ensure that the rights of subjects are protected.

- Monitoring study conduct to ensure compliance with protocol and GCP guidelines.
- Monitoring accuracy, completion, and verification from source documents of study data.

Monitoring of the study conduct will be ongoing. During the study, the monitor will have regular contacts with the investigational site and will be available between visits if the Investigator or other staff needs information or advice.

11.2. Audits and Inspections

To ensure compliance with GCP and all applicable regulatory requirements, the Sponsor may conduct a QA audit.

Authorized representatives of the Sponsor, a regulatory authority, or an IRB may visit the site to perform audits or inspections, including source data verification. The purpose of a Sponsor audit or inspection is to systematically and independently examine all study-related activities and documents to determine whether these activities were conducted, and data were recorded, analyzed, and accurately reported according to the protocol, GCP guidelines of the ICH, and any applicable regulatory requirements. The Investigator should contact the Sponsor immediately if contacted by a regulatory agency about an inspection.

11.3. Case Report Forms and Study Records

The Investigator must maintain source documents for each subject in the study and for all data collected. All information in the eCRFs must be traceable to these source documents, which are generally maintained in the subject's file. The source documents should contain all demographic and medical information as well as a copy (with original signature) of the informed consent provided by the subject. All study data collected in or generated at the study center will be accurately recorded on source documents by qualified research personnel. Site personnel will exercise due diligence in ensuring that study data are entered accurately and in their entirety from the site's source documents into the appropriate eCRF data fields after each subject's visit.

Although the study eCRF is part of the primary database for the study, all data entered into the eCRF must be recorded in the source documents, and any missing data must be explained. Source data will be retained by the site as described in Section 12.2.3.

Only staff designated by the Investigator on the "Delegation of Authority" form in the regulatory binder will be eligible to enter or make edits to the data. Only the Investigator will be authorized to electronically sign the eCRFs.

11.4. Protocol Deviations

No departure from the approved protocol and procedures is permitted for any reason except subject safety, and in that case, the Investigator (or designee) will promptly notify the Sponsor. The IRB will also be informed in a timely manner, consistent with their requirements.

A protocol deviation occurs when there is any non-adherence to a study procedure or schedule that is specified by the protocol. The term "protocol deviation" includes those departures from the protocol previously described by the term "protocol violation"; all departures from the protocol are now described as protocol deviations, regardless of the potential impact on subject safety.

Protocol deviations will be summarized in the final CSR.

12. ADMINISTRATIVE CONSIDERATIONS

12.1. Ethics

12.1.1. Ethics Review Board Approval

The Investigator will provide the ethics review board (e.g., IRB) with this protocol, the ICF, all written material to be provided to the subject, and all advertisements that may be used for subject recruitment. No subjects will be enrolled in the study until the IRB provides written approval of the protocol, any amendments, and the ICF, and until approval documents have been obtained by the Investigator and copies received by the Sponsor. Appropriate reports on the progress of this study by the Investigator will be made to the IRB and the Sponsor in accordance with the applicable government regulations and in agreement with policy established by the Sponsor.

The Investigator is responsible for informing the IRB of any amendment to the protocol, in accordance with local requirements. Progress reports and notifications of serious ADR will be provided to the IRB according to local regulations and guidelines.

The Investigator is also responsible for providing the IRB with reports of any reportable serious ADRs from any other studies conducted with the investigational product. The Sponsor will provide this information to the Investigator.

12.1.2. Ethical Conduct of the Study

This study will be conducted in accordance with GCP requirements described in the current revision of ICH Guidelines and all applicable regulations, including the current CFR Title 21, Parts 50, 54, 56, and 312, and Title 45, Part 164. Compliance with these regulations and guidelines also constitutes compliance with the ethical principles described in the current revision of the Declaration of Helsinki. This study will also be carried out in accordance with local legal requirements.

12.1.3. Subject Information and Consent

The Investigator will ensure that the subject is given full and adequate oral and written information about the nature, purpose, possible risk and benefit of the study. Subjects must also be notified that they are free to discontinue from the study at any time. The subject should be given the opportunity to ask questions and allowed time to consider the information provided.

A properly executed, written ICF, in compliance with FDA regulations and GCP guidelines, shall be obtained from each subject prior to performing any protocol-related procedures that would not otherwise be required for the care of the subject. A copy of the ICF to be used will be submitted by the Investigator to the IRB for review and approval prior to the start of the study. The Sponsor will approve the ICF and all amendments prior to submission to the IRB. The Investigator shall provide a copy of the signed ICF to the subject and a copy shall be maintained in the subject’s medical record.

12.1.4. Confidentiality

All information provided to the Investigator by the Sponsor or their designees, including data in the Investigator’s Brochure, protocols, eCRFs, and verbal and written information will be kept strictly confidential and confined to the clinical personnel involved in conducting the study. It is recognized that this information may be released in confidence to the IRB. In addition, no reports or information about the study or its progress will be provided to anyone not involved in the

study other than to the Sponsor or its designees, or in confidence to the IRB, except if required by law.

12.2. Data Handling and Recordkeeping

12.2.1. Direct Access to Source Data/Documents

Source data are all original records of clinical findings, observations, or other activities in a clinical study which are necessary to achieve the study objectives and protect subject safety. Source data are contained in source documents. Examples of these original documents, and data records include: hospital records, clinical and office charts, laboratory notes, memoranda, subjects' workbooks, diaries, or evaluation checklists, pharmacy dispensing records, recorded data from automated instruments, copies or transcriptions certified after verification as being accurate and complete, microfiches, photographic negatives, microfilm or magnetic media, X-rays, subject files and records kept at the pharmacy, at the laboratories, and at medicotechnical departments involved in the clinical study.

Source documents are the originals of any document used by the Investigator or hospital/institution that allows verification of the existence of the subject and substantiates the integrity of data collected during the study.

Source documents will be available to support all the data recorded in the eCRF. The Investigator must allow designated representatives of the Sponsor and regulatory inspectors to have direct access to the source documents to verify the data reported in the eCRFs.

12.2.2. Inspection of Records

The Sponsor will be allowed to conduct site visits to the investigation facilities for the purpose of monitoring any aspect of the study. The Investigator agrees to allow the monitor to inspect the drug storage area, study drug inventory, drug accountability records, subject charts and study source documents, and other records relative to study conduct.

12.2.3. Retention of Records

In accordance with GCP guidelines, all study-related documentation shall be retained by the Investigator for at least the minimum time required by applicable law for the USA, a minimum of 2 years after marketing approval of GTX-104 or withdrawal of the Sponsor's Investigational New Drug, IND (CFR Title 21, Part 312.57). At that time, the Investigator will contact the Sponsor regarding further disposition of the study records and comply with the Sponsor's instructions.

The Investigator agrees to adhere to the document retention procedures by signing the study protocol. Examples of essential documents include, but are not limited to:

- IRB correspondence indicating approval for the study protocol, ICF(s), and all amendments to either of these documents
- All source documents and laboratory records
- ICFs signed by the subject
- Completed Form FDA 1572, Statement of Investigator
- Any other pertinent study document

12.3. Financial Disclosure

Clinical investigators are required to provide financial disclosure information to allow the Sponsor to submit the complete and accurate certification or disclosure statements required under CFR Title 21, Part 54. As defined in subpart 54.2, a clinical investigator is a listed or identified investigator or sub-investigator who is directly involved in the treatment or evaluation of research subjects. The term also includes the spouse and each dependent child of the Investigator.

In addition, investigators must promptly update this financial disclosure information if any relevant changes occur during the course of the investigation and for 1 year following completion of the study.

12.4. Publication and Disclosure Policy

Following completion of the study, the data from the entire study or from subsets of the study may be considered for reporting at a scientific meeting or for publication in a scientific journal, in which case the Sponsor will be responsible for these activities and will work with the investigators to determine how the manuscript is written and edited, the number and order of authors, the publication to which it will be submitted and other related issues.

13. REFERENCES

Abboud T, Regelsberger J. Serum Levels of Nimodipine in Enteral and Parenteral Administration in Patients with Aneurysmal Subarachnoid Hemorrhage. *Acta neurochirurgica.* Jul 2015;157(7):1133-1134.

Adnet P, Fesard P, Riegel B, Debout J, Krivosic-Horber R. [Interaction of nimodipine and isoflurane in surgery of intracranial aneurysm]. *Agressologie: revue internationale de physio-biologie et de pharmacologie appliquees aux effets de l'agression.* Jun 1989;30(7):399-401.

Allen GS, Ahn HS, Preziosi TJ, et al. Cerebral arterial spasm--a controlled trial of nimodipine in patients with subarachnoid hemorrhage. *The New England journal of medicine.* Mar 17 1983;308(11):619-624.

Auer LM, Oberbauer RW, Schalk HV. Human pial vascular reactions to intravenous Nimodipine-infusion during EC-IC bypass surgery. *Stroke.* Mar-Apr 1983;14(2):210-213.

Barker FG, 2nd, Ogilvy CS. Efficacy of prophylactic nimodipine for delayed ischemic deficit after subarachnoid hemorrhage: a metaanalysis. *Journal of neurosurgery.* Mar 1996;84(3):405-414.

Bioavailability Studies Submitted in NDAs or INDs — General Considerations: FDA Guidance for Industry. 2019.

Boldt J, Von Bormann B, Kling D, Ratthey K, Hempelmann G. Influence of nimodipine and nifedipine on intrapulmonary shunting--a comparison to other vasoactive drugs. *Intensive care medicine.* 1987;13(1):52-56.

Dalal PM, Dalal KP. Use of calcium channel blockers in acute ischemic cerebrovascular disease. *The Journal of the Association of Physicians of India.* Jun 1995;43(6):394-397.

Diringer MN, Bleck TP, Claude Hemphill J, 3rd, et al. Critical care management of patients following aneurysmal subarachnoid hemorrhage: recommendations from the Neurocritical Care Society's Multidisciplinary Consensus Conference. *Neurocritical care.* Sep 2011;15(2):211-240.

Dorhout Mees SM, Rinkel GJ, Feigin VL, et al. Calcium antagonists for aneurysmal subarachnoid haemorrhage. *Cochrane Database Syst Rev.* Jul 18 2007(3):Cd000277.

Gelmers HJ. Effect of nimodipine (Bay e 9736) on postischaemic cerebrovascular reactivity, as revealed by measuring regional cerebral blood flow (rCBF). *Acta neurochirurgica.* 1982;63(1-4):283-290.

Gomis P, Graftieaux JP, Sercombe R, Hettler D, Scherpereel B, Rousseaux P. Randomized, double-blind, placebo-controlled, pilot trial of high-dose methylprednisolone in aneurysmal subarachnoid hemorrhage. *Journal of neurosurgery.* Mar 2010;112(3):681-688.

Harders A, Kakarieka A, Braakman R. Traumatic subarachnoid hemorrhage and its treatment with nimodipine. German tSAH Study Group. *Journal of neurosurgery.* Jul 1996;85(1):82-89.

Ma JJ, Yang S, Wei W. A phase II clinical evaluation of fasudil hydrochloride for cerebral vasospasm following subarachnoid hemorrhage. *Chinese Journal of Neurosurgery*. 2009;2006-01.

Muller H, Marck P, Gips H, et al. [Effect of the calcium antagonist nimodipine on hemodynamics, gas exchange and endocrine parameters in opiate anesthesia]. *Der Anaesthesist*. Oct 1987;36(10):561-569.

Nimodipine Capsules for Oral Use. Drug Label Information. *Heritage Pharmaceuticals Inc., Eatontown, NJ*. August, 2012.

NIMOTOP (0.02% Solution for Infusion) Summary of Product Characteristics. *Bayer PLC, Newbury*. July 2016.

NIMOTOP® (nimodipine) Capsules for Oral Use. FDA approved Labeling text, NDA 18 869 014. *Bayer Pharmaceuticals Corp., West Haven, CT*. 2005.

NYMALIZE™ (nimodipine) oral solution. Highlights of Prescribing Information. *Arbor Pharmaceuticals Inc., Atlanta, GA*. 2013.

Pickard JD, Murray GD, Illingworth R, et al. Effect of oral nimodipine on cerebral infarction and outcome after subarachnoid haemorrhage: British aneurysm nimodipine trial. *BMJ (Clinical research ed.)*. Mar 11 1989;298(6674):636-642.

Rinkel GJ. Management of patients with aneurysmal subarachnoid haemorrhage. *Curr Opin Neurol*. Feb 2016;29(1):37-41.

Rowland MJ, Hadjipavlou G, Kelly M, Westbrook J, Pattinson KT. Delayed cerebral ischaemia after subarachnoid haemorrhage: looking beyond vasospasm. *British journal of anaesthesia*. Sep 2012;109(3):315-329.

Shibao C, Lipsitz LA, Biaggioni I. ASH position paper: evaluation and treatment of orthostatic hypotension. *Journal of clinical hypertension (Greenwich, Conn.)*. Mar 2013;15(3):147-153.

Soppi V, Kokki H, Koivisto T, et al. Early-phase pharmacokinetics of enteral and parenteral nimodipine in patients with acute subarachnoid haemorrhage - a pilot study. *European journal of clinical pharmacology*. Apr 2007;63(4):355-361.

Vinge E, Andersson KE, Brandt L, Ljunggren B, Nilsson LG, Rosendal-Helgesen S. Pharmacokinetics of nimodipine in patients with aneurysmal subarachnoid haemorrhage. *European journal of clinical pharmacology*. 1986;30(4):421-425.

Wanner-Olsen H, Gaarskaer FB, Mikkelsen EO, Jakobsen P, Voldby B. Studies on concentration-time profiles of nimodipine enantiomers following intravenous and oral administration of nimodipine in patients with subarachnoid hemorrhage. *Chirality*. Oct 2000;12(9):660-664.

Whelton PK, Carey RM, Aronow WS, et al. 2017 ACC/AHA/AAPA/ABC/ACPM/AGS/APhA/ASH/ASPC/NMA/PCNA Guideline for the Prevention, Detection, Evaluation, and Management of High Blood Pressure in Adults: Executive Summary: A Report of the American College of Cardiology/American Heart Association Task Force on Clinical Practice Guidelines. *Hypertension (Dallas, Tex. : 1979)*. Jun 2018;71(6):1269-1324.

Zhang P, Wang B, Wang E, Chen X. Preliminary study on controlled hypotension induced by nimodipine in craniocerebral surgery. *Chinese medical journal.* Jun 1995;108(6):420-422.

APPENDICES

Appendix 1. Estimated Volume of Blood for Each Subject

Test	Number of Samples	Volume per Sample (mL)	Total Volume (mL)
Serum Chemistry [a]	3	8.5	25.5
Hematology<br>Hb A1c	3	4.0	12
Coagulation	3	2.7	8.1
PK Samples	116[b]	3	348
**Estimated Total Blood Volume (Overall Study)**			**393.6**

FSH = follicle-stimulating hormone; HbA1c = hemoglobin A1c; PK = pharmacokinetics

[a] Serology, pregnancy testing, and FSH testing will be conducted with the blood sample collected for serum chemistry.

[b] 58 for oral and IV, respectively.

Note: Additional blood samples may be drawn at the discretion of the Investigator (e.g., to retest out-of-range laboratory values or to follow a laboratory AE to resolution).

Appendix 2. Management of Heart Rate and Blood Pressure Decreases

**Document as an AE (or SAE, if applicable) if any of the following criteria are met:**	**Consider a DOSE HOLD if any of the following criteria are met after evaluation by PI:**	**Clinical Interventions:**
If the subject shows symptoms of hypotension and/or bradycardia (e.g., lightheadedness, dizziness, syncope, signs of hypoperfusion) regardless of the vital sign assessment.	If the subject shows symptoms of hypotension and/or bradycardia (e.g., lightheadedness, dizziness, syncope, signs of hypoperfusion) regardless of the vital sign assessment.<br><br>If the following vital sign assessments are observed pre-dose:<br>Resting (sitting or recumbent if necessary due to an AE):<br>• SBP <90 mmHg and >20 % below baseline value;<br>• DBP <50 mmHg and >20% below baseline value; or<br>• Heart rate <50 beats per minute and >20% below baseline value.<br>Orthostatic (after standing for 3 minutes):<br>• SBP, DBP, or heart rate >25% below sitting/recumbent values.	**For all subjects:** Inform subjects that nimodipine may cause hypotension and that moving from a supine to an upright position may increase risk for hypotension and orthostatic effects. Encourage subjects to stay hydrated. Offer fluids frequently. Monitor for symptoms of low blood pressure.<br><br>**For all subjects with asymptomatic or symptomatic hypotension or who otherwise meet the dose hold criteria:** Place subject on bed rest and encourage to drink fluids.<br><br>**For asymptomatic blood pressure and heart values that meet one of the dose hold criteria:** Repeat the vital sign assessment approximately 10 minutes later and if confirmed, hold the dose. If not confirmed with second assessment, take a third set of vital signs approximately 10 minutes later and hold the dose if confirmed in 2 of the 3 assessments.<br><br>**For syncope or severe symptomatic hypotension:** Monitor vital signs at least every 15 minutes until the subject is stable and able to resume normal activities.<br><br>Discontinue subject from study if any of the following criteria are met:<br>• Persistent signs or symptoms of hypotension, bradycardia, or hypoperfusion.<br>• Single occurrence of symptomatic bradycardia (as assessed by the Investigator, regardless of blood pressure) associated with chest pain, shortness of breath, or decreased level of consciousness.<br>• Any other clinically significant cardiovascular signs or symptoms that would place the subject at risk.

AE = adverse event; DBP = diastolic blood pressure; SAE = serious adverse event; SBP = systolic blood pressure

Appendix 3. Summary of Changes from Original Version to Amendment 1

Amendment	Section	Changes	Rationale
Amendment 1	Appendix 3: Summary of Changes	Section added	Section was added to allow description of changes for the first amendment.
	• Synopsis, Section 4.1, Section 4.3, Section 6.3<br><br>Section 1.3.2	The test treatment (Treatment A) dose of GTX-104 was changed from a once daily loading dose to administration of 3.75 mg GTX-104 every 4 hours (q4h) with continuous infusion of 0.2 mg/h.<br><br>The dose justification was updated accordingly with modeling to describe the expected correlation between GTX-104 with this dosage and oral nimodipine at the dose level of 60 mg q4h for 72 hours.	The dosing schedule was changed to more closely mimic the peak/trough pattern of oral nimodipine, which is administered q4h.<br>Based on popPK modeling and simulations, the GTX-104 IV dose regimen of a 3.75 mg q4h dose over 30 minutes in conjunction with 0.2 mg/h continuous infusion is likely to match C_{max} and AUC for oral nimodipine 60 mg q4h, as well as the shape of the overall concertation-time profiles, particularly the 6 peaks within a day corresponding to the q4h schedule for both IV and oral regimens.
	Synopsis, Section 4.1, Table 4-1, Section 8.11, Appendix 1	The PK sampling schedule for GTX-104 was updated and the number of PK samples and overall blood volume was updated accordingly.	The PK sampling schedule was updated to reflect the new sampling regimen.
	• Synopsis, Section 4.1, Section 4.3, Section 8.5, Section 8.10	BP measurement by oscillometric sphygmomanometer was updated to clarify that the q15 minute measurements for the first hour should be taken after each oral dose or 3.75 mg dose of infusion.	BP monitoring was updated to be recorded prior to first dose and 60 minutes post dose for 8:00 AM, 12:00 PM, 4:00 PM, and 8:00 PM doses, for both Treatment A and Treatment B on Days 3 and 10 only.
		An interim PK data analysis section was added.	
		The sample size determination was updated.	

Appendix 4. Summary of Changes from Amendment 1 to Amendment 2

Amendment	Section	Changes	Rationale
Amendment 2	Appendix 4: Summary of Changes	Section added	Section was added to allow description of changes for the first amendment.
	Synopsis, Section 1.3, Section 1.3.2, Section 3.1, Section 4.3, Section 6.2, Section 6.3, Section 10.5.2, Table 1-1, Table 4-1, Figure 1-1 through Figure 1-3, Figure 4-2	Revision to doses and schedules and description of new simulations for dose rationale and justification; Update to PK analyses based on these revisions; Clarification of primary endpoints based on these revisions	Doses and schedules were revised in order to address FDA feedback on endpoints.
	Section 1.2.3, Section 4.1, Section 10.5.1	PK interim analysis stated that it “may be conducted” rather than “is planned”.	A decision will be made after enrollment and treatment has begun whether to perform an interim analysis.
	Synopsis, Section 4.1, Section 8.1	Revision to PK blood sampling schedule	Schedule was revised to accommodated adjustments to revisions to the doses and dose administration schedule.
	• Synopsis, Section 4.1, Section 4.3.3.1, Section 8.5, Section 8.10, Section 8.14, Table 4-1	Revised schedule for blood pressure determination	Schedule was revised to be more accommodating logistically for the CRU.
	Synopsis, Section 4.1, Section 10.2	Updated sample size calculations to plan to enroll a minimum of 60 subjects with at least 50 completing.	Revised enrollment numbers based on study size and power calculations which incorporated data from newly performed PK simulations an to incorporated endpoints based on FDA feedback.
	Synopsis, Section 4.1, Section 4.1.2, Table 4-1	Revised water restrictions	Previous restrictions were overly burdensome for subject and relaxing removed some risk of hypotension.

	Synopsis, Section 4.1, Section 4.1.2, Section 4.3.1, Section 5.1, Section 5.2, Section 8.9, Table 4-1, Table 8-1	COVID-19 testing added	Required given risk of transmission.
	Table 4-1	Added confirmation of eligibility at the start of Treatment 2	To confirm subjects remain eligible throughout both admissions, with considerations for COVID-19.
	Synopsis, Section 5.2	Removed the stated requirement "Does not meet allincludion criteria" from the inclusion criteria.	Condition removed as redundant.
	Table 4-1	Removed scheduled ECGs between Screening and EOS/ET	ECGs during treatment judged to be unnecessary for healthy subjects being administered an approved drug.
	Section 6.6.1	Clarification of birth control restrictions for subjects at Screening.	Text revised to clarify requirements for birth control use prior to Screening if Screening occurs less than 30 days prior to the initial CRU admission.
	Synopsis, Section 3.2	Specified that secondary endpoints "will include:", revised from "may include:"; Addition of Cmax on Day 3 across all 6 doses as a secondary endpoint.	Endpoints were revised to address FDA feedback.
	Appendix 1	Blood collection volume for PK sampling and total estimated blood collection volume per subject revised down.	Blood collection volume for PK sampling was revised to reduce total volume of blood required to be collected from each subject over the course of the study.

Appendix 5. Summary of Changes from Amendment 2 to Amendment 3

Amendment	Section	Changes	Rationale
Amendment 3	5: Summary of Changes	Section added	Section was added to allow description of changes for the amendment.
	Title Page	Added PI and medical monitor name and address	Based on CRO selection
	Synopsis, Section 3.2,	Revision to secondary objectives	since intermediate or poor metabolizers CYP3A4 are to be excluded.
	Synopsis	Updated country of the trial	Based on CRO selection
	Section 4.1, Section 5.0	Clarified wording for sample size calculations to plan to enroll a minimum of 60 subjects with at least 50 completing.	Revised enrollment numbers based on study size and power calculations which incorporated data from newly performed PK simulations an to incorporated endpoints based on FDA feedback.
	Section 4.1.2,	Revised timing for COVID-19 virus test	To meet the testing guidelines of CRO
	Section 4.1.2	Revised language in footnotes f and J	Text revised to clarify requirements
	Section 4.3.2, Section 8.5	Revised type of thermometer to be used, added temperature in Celsius scale	Text revised to clarify requirements, country specific requirements
	Section 5.1	Split the inclusion criteria in two separate criteria<br>Revised wording<br>Added serology test	Text revised to clarify inclusion requirement<br>Text revised to clarify who can make decision<br>To screen patient with any abnormal tests
	Section 5.2	Revised wording in criteria 11and 16	Text revised to clarify requirements
	Section 6.1.1	Revised wording	Text revised to clarify who can make decision
	Section 7.1.1	Added French as requirement for IP label	Text revised to clarify based on CRO site
	Section 7.3	Revised wording	Text revised to clarify who can make decision

	Section 8.2	Updated wording for subject identification number	To meet the procedures and guidelines of CRO
	Section 10.5.1	Added wording for additional number of subjects to be enrolled	Based on interim pkanalysis number of subjects will be adjusted
	Synopsis, Section 4.1, Section 4.3.1, Section 5.25.2	Changed screening window from 28 days to 45 days	To screen sufficient number of subjects needed for trial
	Synopsis, Abbreviations, Section 4.1	Defined Reference Listed Drug and Reference Substances	To differentiae between Reference listed drug and available reference standard to be used in the study.

Appendix 6. Summary of Changes from Amendment 3 to Amendment 4

Amendment	Section	Changes	Rationale
Amendment 4	Summary of Changes	Section added	Section was added to allow description of changes for the f amendment.
	TOC, Section4.1.2.Table4.1, Section 4.5	Changed screening window from 28 days to 45 days	Inadvertently was missed in earlier amendment..
	Synopsis	Updated Inclusion Exclusion Criteria	Based on wording in the main body of the protocol

Appendix 7 Summary of Changes from Amendment 4 to Amendment 5

Amendment	Section	Changes	Rationale
Amendment 5	Synopsis	Participant duration in the study was changed from ~40 days to ~56 days	Based on extended screening window
	Synopsis Inclusion criteria 13, Section 5.1, section 8.8	Added blood test for genetic testing to identify metabolic genotype for CYP3A4	Due to on going Covid-19 pandemic buccal swabs are in short supply and thus blood test is added
	Synopsis Inclusion criteria 17, section 5.1	Restriction on sperm donation for male subjects reduced to 30 days from 90 days to match with criteria15	Based on literature information for nimodipine and similar class of compounds it was too restrictive

	Section 7.1.2	Updated capsule in print to NM1 from H108	Typographical error corrected to match the description on RS capsules
	Section 8.15.3	Updated the wording and symbol	To clarify the time the action needs to be taking for unplanned infusion interruptions and to match with table 8.2

Appendix 8 Summary of Changes from Amendment 5 to Amendment 6

Amendment	Section	Changes	Rationale
Amendment 6	Title page and footer of the document	Updated the version and date of amendment.	Requested by CRO (Altasciences) due to Amendment 05 of the protocol being submitted to the IRB prior to receiving the NOL from Health Canada.
	Synopsis, Section 4.1.2 Table 4.1 footnote J	Added serology test at screening only<br>Added Day 4 for Covid testing	To clarify when serology test is to be performed<br>In order to be consistent with exclusion criterion 2
	Synopsis, Section 5.2	Revised eclusion criteria 20 from 90 days to 30 days	For consistency with inclusion criteria 17
	Table 6.1, Section 8.3	Corrected requirement for Experimental drug and/or experimental medical device from 3 months to 28 days	For consistency throughout the protocol as described in exclusion criterion 16
	Section 8.11.1.1	Added 0.5 timepoints, blood samples for PK analysis	For consistency throughout the protocol as described in Table 4.1
	Section 8.15.3 and Table 8.2	Added language for how to manage infusion interruption	To clarify how interruption in infusion will be managed.
	Table 8.1	Deleted Covid test under serology	Was indadvertently included under serology
